# Ubiquitin-Like protein 5 interacts with the silencing suppressor p3 of rice stripe virus and mediates its degradation through the 26S proteasome pathway

**DOI:** 10.1371/journal.ppat.1008780

**Published:** 2020-08-31

**Authors:** Binghua Chen, Lin Lin, Yuwen Lu, Jiejun Peng, Hongying Zheng, Qiankun Yang, Shaofei Rao, Guanwei Wu, Junmin Li, Zhuo Chen, Baoan Song, Jianping Chen, Fei Yan

**Affiliations:** 1 Center for Research and Development of Fine Chemicals, Guizhou University, China; 2 State Key Laboratory for Managing Biotic and Chemical Threats to the Quality and Safety of Agro-products, Key Laboratory of Biotechnology in Plant Protection of MOA of China and Zhejiang Province, Institute of Plant Virology, Ningbo University, China; 3 Institute of Virology and Biotechnology, Zhejiang Academy of Agricultural Sciences, Hangzhou, China; Institute of Microbiology, CHINA

## Abstract

Ubiquitin like protein 5 (UBL5) interacts with other proteins to regulate their function but differs from ubiquitin and other UBLs because it does not form covalent conjugates. Ubiquitin and most UBLs mediate the degradation of target proteins through the 26S proteasome but it is not known if UBL5 can also do that. Here we found that the UBL5s of rice and *Nicotiana benthamiana* interacted with rice stripe virus (RSV) p3 protein. Silencing of *NbUBL5s* in *N*. *benthamiana* facilitated RSV infection, while *UBL5* overexpression conferred resistance to RSV in both *N*. *benthamiana* and rice. Further analysis showed that NbUBL5.1 impaired the function of p3 as a suppressor of silencing by degrading it through the 26S proteasome. NbUBL5.1 and OsUBL5 interacted with RPN10 and RPN13, the receptors of ubiquitin in the 26S proteasome. Furthermore, silencing of *NbRPN10* or *NbRPN13* compromised the degradation of p3 mediated by NbUBL5.1. Together, the results suggest that UBL5 mediates the degradation of RSV p3 protein through the 26S proteasome, a previously unreported plant defense strategy against RSV infection.

## Author summary

The ubiquitin-mediated 26S proteasome has been reported to inhibit viral infection in plants. We here found that the 26S proteasome also degraded the p3 suppressor of RNA silencing of rice stripe virus to inhibit viral infection, and that the degradation was mediated by ubiquitin-like protein 5 (UBL5) but not by ubiquitin. UBL5 has been reported to modulate the proteins with which it interacts by being part of a functional complex, but not by directly mediating its degradation. Our results suggest that UBL5 may also play a role in mediating target protein degradation, a previously unreported function and one that it shares with ubiquitin. This is also the first report that UBL5 interacts with a pathogen protein and that UBL5-mediated regulation might also participate in plant defense against pathogens.

## Introduction

Ubiquitin is a 76-residue (8.6 kDa) regulatory protein that is conserved throughout the eukaryotic kingdom [1, 2]. Attachment of ubiquitin to cellular proteins (referred to as ubiquitination or ubiquitylation) changes the stability, localization, or activity of the target protein [1]. Depending on the ubiquitin chain types used for the ubiquitination, proteins either get targeted to the proteasome for degradation or acquire new binding surfaces that can alter their subcellular localization and function[3, 4]. Ubiquitin binds to target proteins via an isopeptide bond between the two glycine (Gly) residues at its C-terminus and an acceptor amino acid of the target protein substrate through the sequential activities of specific enzymes: ubiquitin activating enzymes (E1), ubiquitin conjugating enzymes (E2), and ubiquitin protein ligases (E3) [1, 5, 6].

A large number of ubiquitin-like proteins (UBLs) have also been found in nearly all higher eukaryotic organisms. UBLs range in size from 73 to 186 amino acids and share a canonical three-dimensional structure called the ubiquitin-fold [3, 7–11]. Many UBLs, such as SUMO (small ubiquitin-related modifier), NEDD8 (neural precursor cell expressed, developmentally down-regulated 8) and ISG15 (interferon-stimulated gene 15), also bind to target proteins for regulation though the sequential activities of an E1-E3 enzymatic cascade [12–14]. Ubiquitin-like protein 5 (UBL5), known as Hub1 in yeast, is somewhat different because it lacks a C-terminal di-glycine motif and is thus unable to form covalent conjugates with other proteins [15, 16]. UBL5/Hub1 is evolutionarily conserved and shares a low degree of sequence similarity with ubiquitin, yet closely resembles it structurally [15, 17–19].

In yeast, Hub1 plays an essential role in pre-mRNA splicing [16, 20–23]. In an early report, Hub1 was found to interact with splicing factor Snu66 and to help it localize correctly at the nucleus for its normal function [20]. Afterwards, structural and biochemical data showed that Hub1 could bind non-covalently to the conserved element termed HIND that is present in Snu66 in yeast and mammals, and Prp38 in plants [21]. Spliceosomes that lacked Hub1, or were defective in their Hub1-HIND interaction, could not use certain non-canonical 5' splice sites, implicating Hub1 in alternative splicing [21]. In addition to Snu66, Hub1 could bind to the DEAD-box helicase Prp5, a key regulator of early spliceosome assembly, and stimulated its ATPase activity [22]. High Hub1 levels enhanced splicing efficiency but also caused mis-splicing by tolerating suboptimal splice sites and branchpoint sequences [22]. Moreover, Prp5 itself was regulated by a Hub1-dependent negative feedback loop [22]. In human cells, UBL5, the orthologue of yeast Hub1, is also essential for pre-mRNA splicing [23–25]. UBL5 depletion decreased pre-mRNA splicing efficiency and led to defective sister chromatid cohesion, which is a general consequence of dysfunctional pre-mRNA splicing [24]. UBL5 also plays roles in the Fanconi anemia (FA) DNA repair pathway and in responses to hypoosmotic conditions [26, 27].

The function of UBL5 in plants has been less well studied but transgenic perennial ryegrass (*Lolium perenne* L.) plants overexpressing UBL5 had improved drought tolerance as shown by greater relative water content, leaf water potential, chlorophyll content and photosynthetic rate when subjected to drought stress [28].

Rice stripe virus (RSV) is an economically important virus of rice in East Asia. It is naturally transmitted by the small brown planthopper (SBPH, *Laodelphax striatellus* Fallén) but it can also infect *Nicotiana benthamiana* by mechanical inoculation and this provides a convenient experimental system for studying virus-host interactions [29, 30]. RSV, the type member of the genus *Tenuivirus* (*Phenuiviridae*, *Bunyavirales*) has a genome of four single-stranded RNAs. p3 protein is encoded by the sense-strand of RNA3 and is a suppressor of RNA silencing [31]. The dimeric p3 can bind to long dsRNA with two or more copies, and the suppression activity of p3 is mechanistically related to its dsRNA binding ability [32, 33]. Expression of p3 induces host resistance by affecting host gene expression in plants [34, 35]. Moreover, by interacting with OsDRB1, an indispensable component of the rice miRNA-processing complex, p3 regulates biogenesis of microRNAs (miRNAs) [36].

Here we found that UBL5 in either *N*. *benthamiana* or rice (*Oryza sativa*) interacted with the RSV p3 protein. Silencing of *UBL5s* in *N*. *benthamiana* facilitated RSV infection, while *UBL5* overexpression conferred resistance against RSV in both *N*. *benthamiana* and rice. We then demonstrate that UBL5 impairs the function of p3 as a suppressor of silencing by degrading it through the 26S proteasome pathway and that UBL5 interacts with the receptors of ubiquitin in the 26S proteasome. This suggests that UBL5 may function by interacting with p3 and delivering it into the 26S proteasome for degradation.

## Results

### *Oryza sativa* UBL5 and *N*. *benthamiana* UBL5s interact with RSV p3

**Fig 1:**
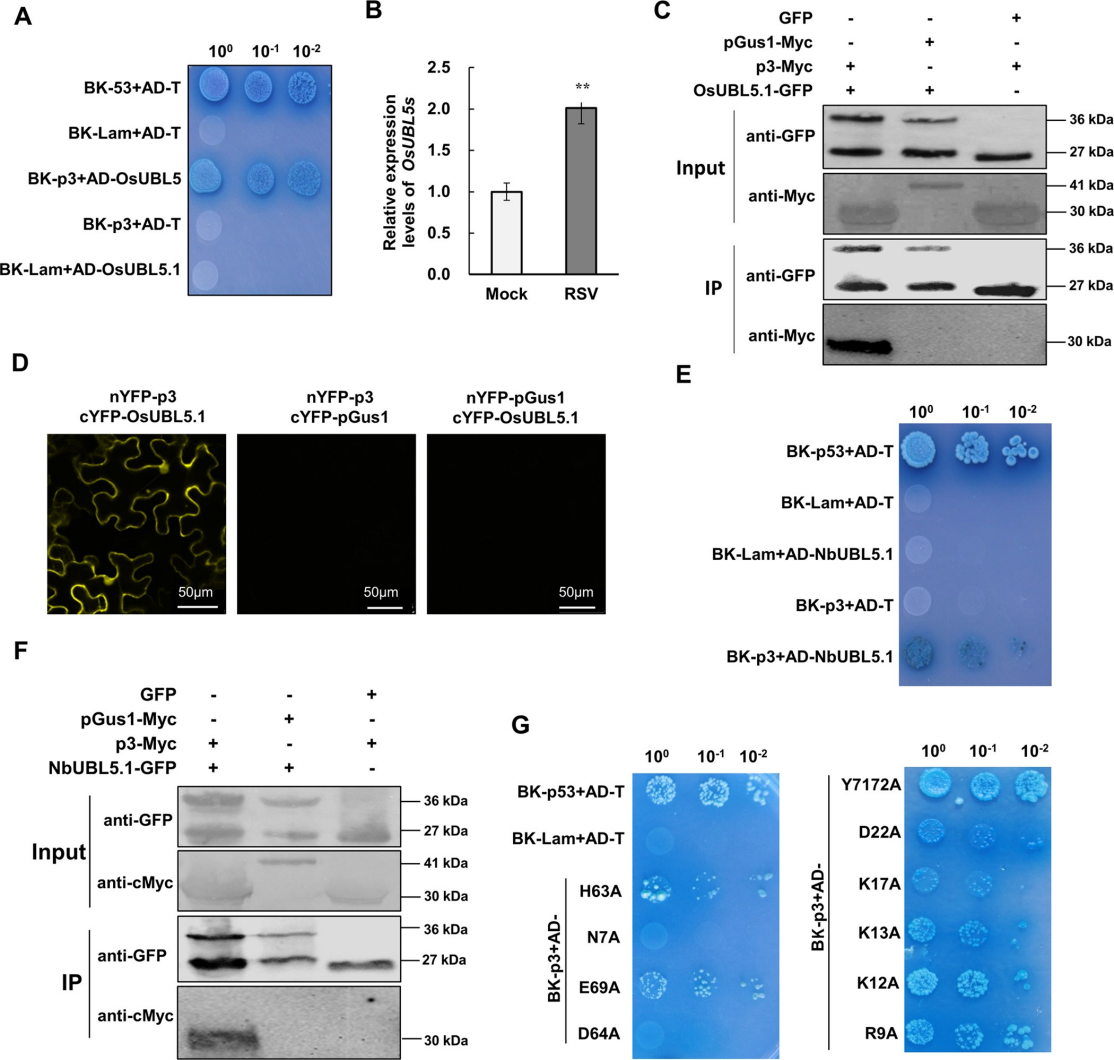
OsUBL5 and NbUBL5.1 interact with RSV p3. A. OsUBL5.1 interacts with RSV p3 protein in a yeast two hybrid (Y2H) assay. Yeast co-transformed with pGBK-p53+pGAD-T served as a positive control, and yeast co-transformed with vectors pGBK-Lam+pGAD-T, pGBK-p3+pGAD-T or pGBK-Lam+pGAD-NbUBL5.1 were the negative controls. Serial 10-fold dilutions of co-transformed yeast cells on synthetic defined (SD) medium (-Ade/-His/-Leu/-Trp) with X-gal are shown. B. In RSV-infected rice plants, the expression of *OsUBL5s* was 2.5-fold greater than in non-infected plants. C. Co-IP demonstrated the interaction between OsUBL5.1 and p3. Co-IP analysis of OsUBL5-GFP and p3-Myc expressed in *N*. *benthamiana* leaves by agroinfiltration. Separate expression of OsUBL5-GFP and p3-Myc, and co-expression of OsUBL5-GFP and pGus1-Myc were used as negative controls. The N-terminal peptide of β-glucuronidase (Gus) consisting of 306 amino acids and named partial Gus protein 1 (pGus1) was used as a non-interacting, negative control. D. BiFC assay demonstrated the interaction between OsUBL5 and p3 in the leaves of *N*. *benthamiana* at 60 h post infiltration (hpi). The N- or C-terminal fragments of YFP were fused to the N-terminus of p3 and OsUBL5. Bars, 50 μm. E and F. Y2H and Co-IP demonstrated the interaction between NbUBL5.1 and p3. G. Detection of interaction between NbUBL5.1 mutants and p3 by Y2H assay showing that mutants R9A and D64A did not interact with p3.

In a yeast two-hybrid (Y2H) assay to screen for rice (*O*. *sativa*) proteins that interacted with RSV p3, *O*. *sativa* UBL5 (XP_015622824.1) gave a positive signal on stringent Leu-, Trp-, His- and Ade- medium (Fig 1A). UBL5 is a member of the Ubiquitin-Like (UBL) Protein subfamily; this sequence of *UBL5* (named *OsUBL5*.*1* here) contains an ORF of 222 nucleotides predicted to encode an 8.5 kDa protein (73 amino acids). A homolog of *OsUBL5*.*1* differing from it by three amino acids (*OsUBL5*.*2*, XP_015616810.1) was also identified in the NCBI database of rice reference proteins. *OsUBL5*.*2* also interacted with p3 (S1 Fig). In RSV-infected rice plants, *OsUBL5s* were expressed 2.5 fold more than in non-infected plants (Fig 1B). The interaction between OsUBL5.1 and p3 was further confirmed by co-immunoprecipitation (Co-IP) and bimolecular fluorescence complementation (BiFC) (Fig 1C and 1D). In the BiFC assay, fluorescence was observed in both the cytoplasm and nucleus, consistent with the reported localization of human UBL5 and RSV p3 [24, 31].

We next investigated whether *N*. *benthamiana* UBL5 also interacted with RSV p3. Three homologs of OsUBL5.1 were identified In *N*. *benthamiana* (https://solgenomics.net/), named NbUBL5.1 (Sequence ID in Sol Genomics Network: Niben101Scf08711g01028.1), NbUBL5.2 (Sequence ID: Niben101Scf03253g00017.1) and NbUBL5.3 (Sequence ID: Niben101Scf01986g00011.1). These sequences have 99% amino acid identity to OsUBL5.1 (S2A Fig). Their expression was upregulated 3.5-fold in RSV-infected *N*. *benthamiana* (S2B Fig). Y2H, BiFC and Co-IP all also confirmed that NbUBL5.1 interacted with RSV p3 (Fig 1E and 1F, S2C Fig). Moreover, NbUBL5.2 and NbUBL5.3 also interacted with RSV p3 in Y2H assays (S2D Fig). These results show that p3 interacts with UBL5 from both *O*. *sativa* and *N*. *benthamiana* and in the following experiments, NbUBL5.1 was used for analysis.

It has been reported that Hub1 contains eleven conserved amino acid sites that are necessary for its function: N7, R9, K12, K13, K17, D22, H63, D64, E69, Y71 and Y72 [21, 22]. We therefore next determined whether these amino acids were essential for the interaction with p3 (S2A Fig). Each of these conserved amino acids was replaced with alanine, mostly in separate mutants (N7A to E69A) but since Y71 and Y72 act jointly [17–19] we replaced them both by alanine in one mutant (Y7172A). In Y2H assays, mutants N7A and D64A did not interact with p3, K17A seemed to have a somewhat weakened interaction but the other mutants interacted normally (Fig 1G). These results were confirmed by BiFC assay (S3 Fig) and suggest that N7 and D64 are essential for the interaction between NbUBL5.1 and RSV p3.

### Silencing of *NbUBL5s* facilitates RSV infection in *N*. *benthamiana*

**Fig 2:**
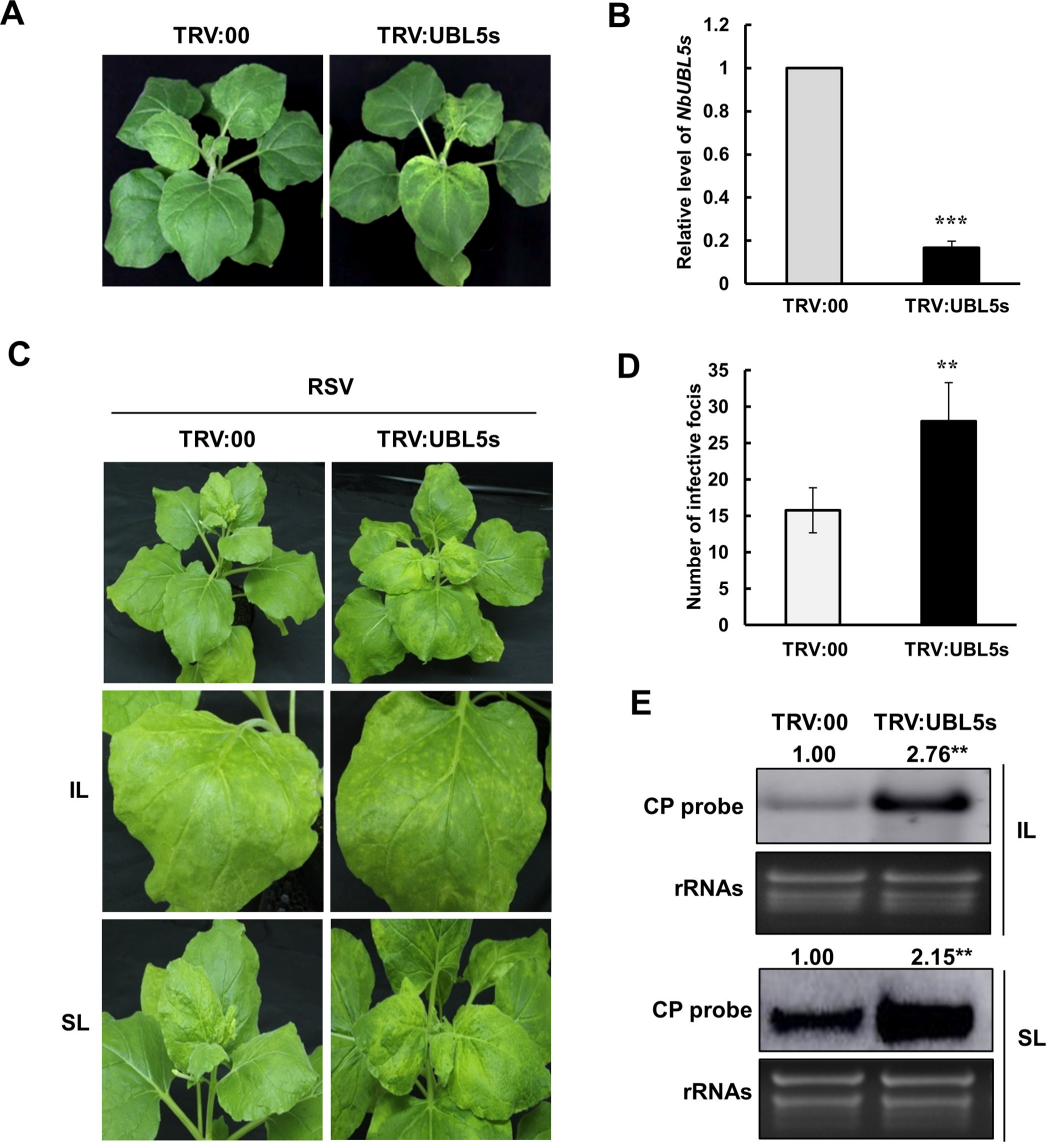
Silencing of *NbUBL5s* facilitates the infection of RSV. A. Silencing of *NbUBL5s* caused chlorosis on the top leaves but had no other effects on plant development. B. qRT-PCR revealed that the expression of *NbUBL5s* in TRV:UBL5s-infected plants was decreased to 20% of that in the control plants treated with TRV:00, indicating the silencing of *NbUBL5s*. C. RSV infection was monitored on *NbUBL5s*-silenced plants. At 7 dpi of RSV inoculation, *NbUBL5s*-silenced plants had more yellow infection foci and higher levels of accumulation of RSV RNAs than controls in their inoculated leaves. At 20 dpi, RSV-systemically infected leaves showed typical RSV symptoms in both TRV:00-infected and TRV:UBL5s-infected plants. D. The numbers of infection foci on the inoculated leaves of plants. E. Northern blot analysis showed that RSV RNAs in *NbUBL5s*-silenced leaves accumulated at higher levels than in controls. RSV CP probe was used for analysis. IL: inoculated leaves. SL: systemically infected leaves. Results are from three independent repeats. Twenty plants were used for each repeat. Bars represent the standard errors of the means from three biological repeats. Band intensity in blots was calculated by ImageJ based on three replicates. A two-sample unequal variance directional t test was used to test the significance of the difference (**, *p* value<0.01; ***, *p* value<0.001).

To investigate the function of UBL5 during RSV infection, we used the tobacco rattle virus (TRV)-induced gene silencing (VIGS) system to silence *NbUBL5s* in *N*. *benthamiana* and challenged the silenced plants with RSV. A sequence of 160 nt from *NbUBL5*.*1* was amplified by PCR and was cloned into the vector TRV2 to yield TRV:UBL5s for use in VIGS. The three *NbUBL5s* have highly similar sequences and so would all be silenced simultaneously in VIGS plants. 12 days after VIGS, silenced (TRV:UBL5s-infected) plants showed chlorosis on the top leaves compared to the controls (TRV:00-infected) but there were no other effects on plant development (Fig 2A). Quantitative RT-PCR (qRT-PCR) showed that the expression of *NbUBL5s* in TRV:UBL5s-infected plants was only 20% of that in the control plants (Fig 2B). TRV RNAs accumulated at similar levels in TRV:UBL5s-infected and TRV:00-infected plants showing that the chlorosis on TRV:UBL5s-infected plants resulted from silencing of *NbUBL5s* and not from differential accumulation of TRV (S4 Fig).

Silenced plants were then mechanically inoculated with RSV. 7 days later, *NbUBL5s*-silenced plants had more yellow infection foci and higher concentrations of RSV RNAs in their inoculated leaves than did the controls (Fig 2C–2E). At 20 dpi, the newly-emerged leaves showed typical symptoms of RSV systemic infection on both TRV:00-infected and TRV:UBL5s-infected plants (Fig 2C). Northern blotting showed that the concentration of RSV RNA in *NbUBL5s*-silenced leaves was double that in control leaves (Fig 2E). The results demonstrate that silencing of *NbUBL5s* facilitated RSV infection.

To minimize any direct effect of the chlorosis shown on *NbUBL5s*-silenced plants on infection by RSV, we used a *NbUBL5*.*1* hairpin RNAi construct as an alternative means of silencing the expression of *NbUBL5*.*1* in leaves, according to the method previously described [37]. Transient expression of the *NbUBL5*.*1* hairpin RNAi construct reduced the expression of *NbUBL5s* to 40% of normal levels, but did not cause obvious chlorosis, probably due to the reduced efficiency of silencing (S5 Fig). At 1 dpi of infiltration of the *NbUBL5*.*1* hairpin RNAi construct, RSV was inoculated onto the leaves. At 7 dpi of RSV, the viral RNAs in *NbUBL5s*-silenced leaves had accumulated to higher levels than those in non-silenced (empty vector-infiltrated) leaves, which indicates that the effect of silencing of *NbUBL5s* on RSV infection was not due to the chlorosis (S5 Fig).

### Transient expression of NbUBL5.1 affects the function of p3 as a suppressor of RNA silencing

**Fig 3:**
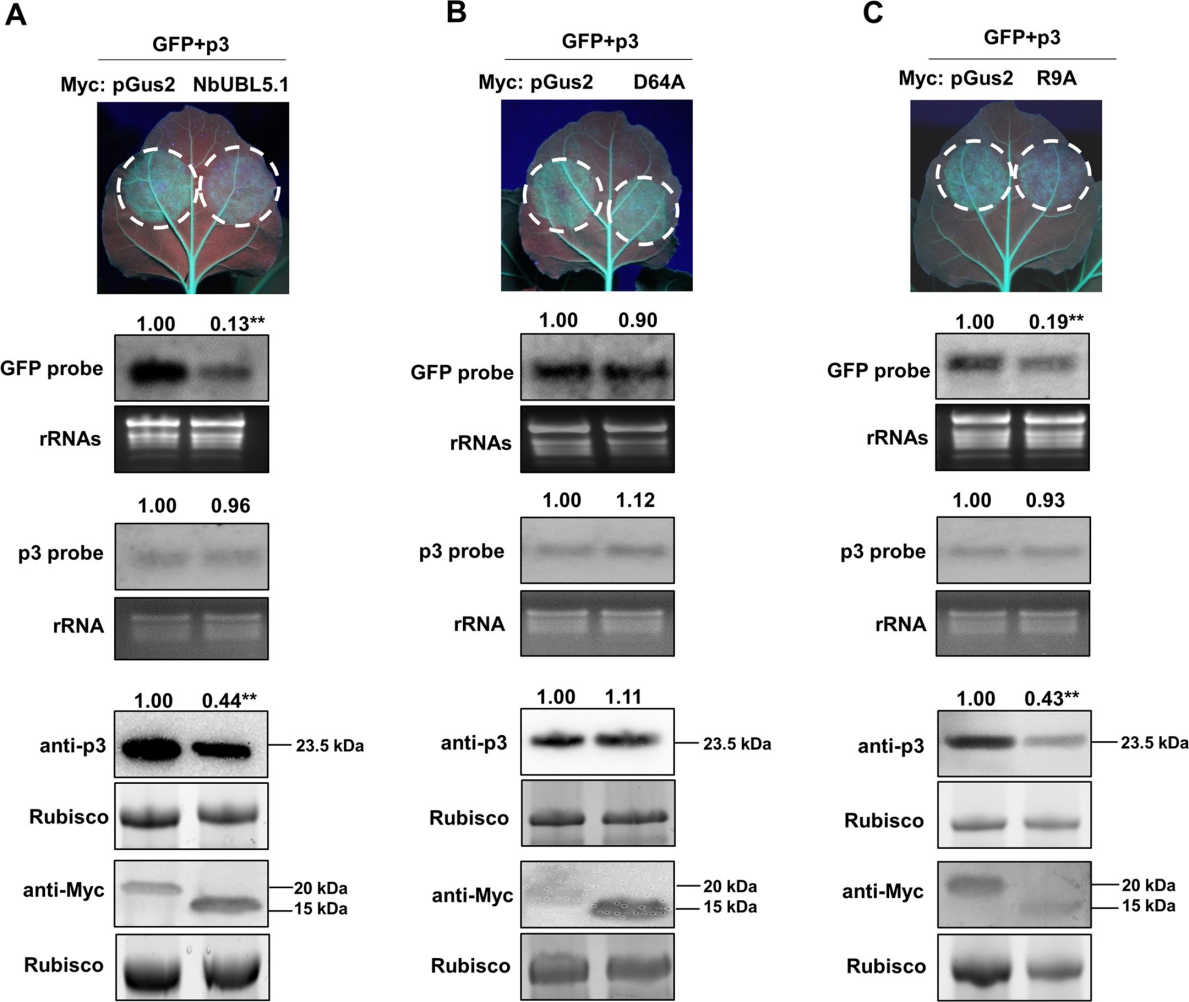
Transient expression of NbUBL5.1 impairs the function of p3 as a suppressor of RNA silencing. A. Myc-fused NbUBL5.1 or control protein pGus2 (The N-terminal peptide of GUS with 122 amino acids) was co-expressed with GFP and p3 in 16c leaves. At 5 dpi, there was strong fluorescence in zones expressing GFP, p3 and pGus2-Myc, while the fluorescence in zones expressing GFP, p3 and NbUBL5.1-Myc was significantly weaker. GFP mRNA accumulated less when NbUBL5.1-Myc was expressed. Western blot demonstrated the expression of Myc-fused pGus2 or NbUBL5.1, while showing that p3 accumulated less in zones expressing Myc-NbUBL5.1, p3 and GFP. B and C. Effect of NbUBL5.1 mutants (D64A and R9A) on p3. Mutant D64A (B) had no effect on p3 accumulation and its function as a silencing suppressor, while mutant R9A (C) retained the effect on p3. Results are from three biological replicates. Band intensity in blots was calculated by ImageJ based on three replicates. A two-sample unequal variance directional t test was used to test the significance of the difference (**, *p* value<0.01).

p3 is the viral suppressor of RNA silencing (VSR) for RSV and plays a key role in successful viral infection [31, 32, 35]. Because silencing of *NbUBL5s* facilitated RSV infection (Fig 2), we next investigated whether the interaction of NbUBL5.1 with p3 affected the VSR function of p3. The assay used leaves of transgenic *N*. *benthamiana* (16c) expressing GFP. When GFP was co-expressed in these leaves with p3 by the agrobacterium infiltration system, the infiltrated zones showed green fluorescence at 5 dpi, but when GFP was expressed with a control protein (N-terminal peptide of β-glucuronidase with 122 amino acids, named pGus2), green fluorescence was absent, thus showing that p3 suppressed the RNA silencing of GFP mRNAs (S6A Fig). Next, Myc-fused NbUBL5.1 or control protein pGus2 was co-expressed with GFP and p3 in 16c leaves. At 5 dpi, there was strong fluorescence in zones expressing GFP, p3 and pGus2-Myc, while the fluorescence in zones expressing GFP, p3 and NbUBL5.1-Myc was significantly weaker (Fig 3A). The accumulation of GFP mRNA in these zones was consistent with the observed fluorescence (Fig 3A). We also tested the effect of NbUBL5.1-Myc on the function of other well-studied VSRs (p19, HC-Pro and 2b) and found that the VSR function of these proteins was not affected by the expression of NbUBL5.1-Myc (S7 Fig). To exclude the possible direct influence of NbUBL5.1 on RNA silencing, we expressed NbUBL5.1-Myc or pGus2-Myc with GFP in 16c leaves. The GFP mRNAs and proteins in the zones expressing NbUBL5.1-Myc and GFP both accumulated at similar levels to those in zones expressing pGus2-Myc and GFP (S6B Fig). These results demonstrate that the co-expression of NbUBL5.1 specifically inhibited the VSR function of p3.

Next, to confirm whether the protein interaction was required for NbUBL5.1 to inhibit the VSR function of p3, we used the NbUBL5.1 mutants R9A and D64A for analysis. Mutant D64A abolished the ability to interact with p3, while mutant R9A did not (Fig 1). When D64A was expressed together with p3 and GFP, fluorescence and accumulation of GFP mRNAs and proteins were at similar levels to those in control zones expressing pGus2, p3 and GFP (Fig 3B), whereas in zones expressing R9A together with p3 and GFP, fluorescence and accumulation of GFP mRNAs and proteins were all significantly reduced compared to those in control zones (Fig 3C). These results indicate that the interaction with p3 was necessary for NbUBL5.1 to inhibit the VSR function of p3.

### NbUBL5.1 mediates p3 degradation through the 26S proteasome system

**Fig 4:**
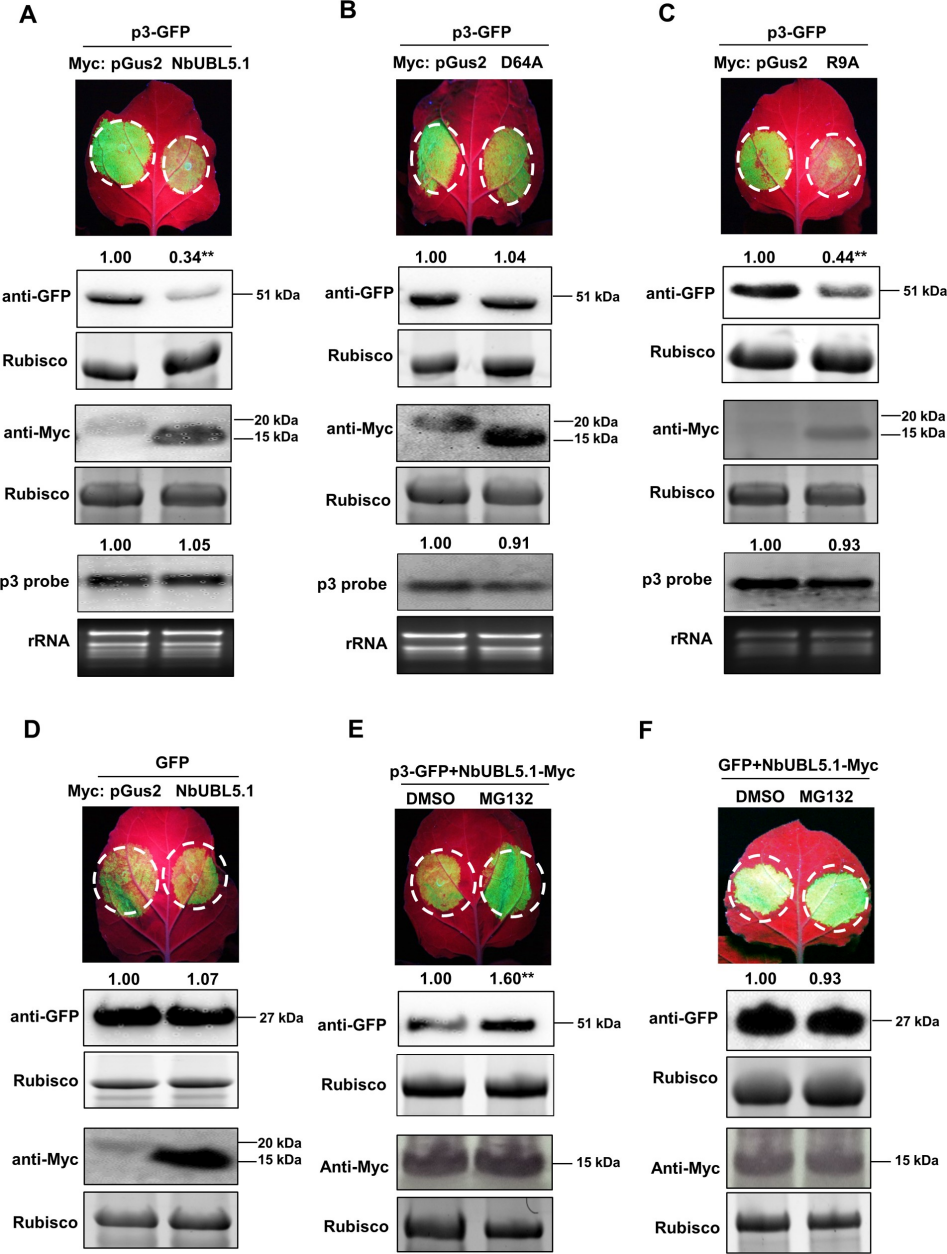
NbUBL5.1 mediates p3 degradation through the 26S proteasome system. A. GFP-fused p3 was co-expressed with Myc-fused NbUBL5.1 or Myc-fused pGus2 as control in leaves of wild type *N*. *benthamiana* through agroinfiltration. At 3.5 dpi, fluorescence and accumulation of GFP-fused p3 protein in zones co-expressing NbUBL5.1-Myc was notably reduced, compared to that in zones expressing pGus2-Myc with p3-GFP. B and C. Effect of NbUBL5.1 mutants, D64A(B) and R9A(C) on p3-GFP accumulation. Fluorescence and accumulation of GFP-fused p3 protein in zones co-expressing R9A-Myc was notably reduced, while those in zones expressing D64A-Myc with p3-GFP were at similar levels to the control. D. Myc-fused NbUBL5.1 did not affect fluorescence and protein accumulation of GFP in the control experiments. E. NbUBL5.1-Myc and p3-GFP were co-expressed in leaves treated with MG132 or DMSO. At 4 dpi, fluorescence and accumulation of GFP-fused p3 protein in zones treated with MG132 were greater than those in zones treated with DMSO. F. MG132 treatment had no effect on the fluorescence and protein accumulation of GFP in the presence of NbUBL5.1-Myc. Expression of the Myc-fused proteins was confirmed by western blot using a Myc antibody. Results are from three biological replicates. Band intensity in blots was calculated by ImageJ based on three replicates. A two-sample unequal variance directional t test was used to test the significance of the difference (**, *p* value<0.01).

While analyzing the effect of NbUBL5.1 on the VSR function of p3 (above), we also measured the expression of p3 and were surprised to find that the accumulation of p3 protein was significantly reduced when it was co-expressed with NbUBL5.1 or mutant R9A but not when co-expressed with mutant D64A (Fig 3A–3C). Meanwhile, transcript levels of p3 were not affected in these experiments (S8A Fig). Therefore, expression of NbUBL5.1 reduced p3 protein and this effect seemed to depend on the interaction between NbUBL5.1 and p3. To confirm this, we next expressed Myc-tagged NbUBL5.1 or its mutants together with GFP-fused p3 in leaves of wild type *N*. *benthamiana*. Fluorescence and protein accumulation of p3-GFP in zones co-expressing NbUBL5.1-Myc or R9A-Myc with p3-GFP were notably less than in zones expressing D64A-Myc with p3-GFP (Fig 4A–4C) whereas the p3-GFP mRNAs in these leaves were at similar levels (S8B Fig). Myc-fused NbUBL5.1 did not itself affect fluorescence and protein accumulation of GFP in the control experiments (Fig 4D). These results further demonstrated that the expression of NbUBL5.1 specifically reduced p3 accumulation.

In the earlier Co-IP assay confirming the interaction between UBL5 and p3, there was no reduction of p3 when co-expressed with GFP-fused OsUBL5 or NbUBL5. We had therefore not at first expected that UBL5 would degrade p3 but now suppose that the fused GFP may affect the normal function of UBL5.

To further demonstrate and confirm the role of UBL5 in degrading p3, we analyzed p3 accumulation in *NbUBL5s*-silenced plants. p3-GFP was expressed in *NbUBL5s*-silenced leaves or non-silenced leaves by infiltration for 3 days, and then the samples were collected for analysis. The results showed that p3-GFP protein did indeed accumulate more in *NbUBL5s*-silenced leaves than in non-silenced leaves (S9 Fig) while at the same time the p3-GFP mRNAs in these leaves were at similar levels (S9 Fig). This provides additional evidence supporting the role of UBL5 in mediating p3 degradation.

The 26S proteasome system is an essential pathway for protein degradation. To know whether it functions in the NbUBL5.1-induced reduction of p3 protein, we inhibited the 26S proteasome system using MG132 and examined the effect on NbUBL5.1-induced reduction of p3 protein. In the control, MG132 treatment inhibited the degradation of tomato yellow leaf curl China virus (TYLCCNV) βC1 protein (reported to be degraded through 26S proteasome system) [38], but did not affect GFP accumulation (S10A and S10B Fig) or the transient expression of NbUBL5.1 (S10C Fig). When NbUBL5.1-Myc and p3-GFP were co-expressed in leaves treated with MG132, the accumulation of p3-GFP was recovered (Fig 4E), while MG132 treatment did not affect GFP accumulation in the presence of NbUBL5.1-Myc (Fig 4F). These indicate that the 26S proteasome pathway was involved in the reduction of p3 induced by NbUBL5.1.

### NbUBL5.1 interacts with the ubiquitin receptors NbRPN10 and NbRPN13 in the 26S proteasome, and their silencing inhibits NbUBL5.1-mediated p3 degradation

**Fig 5:**
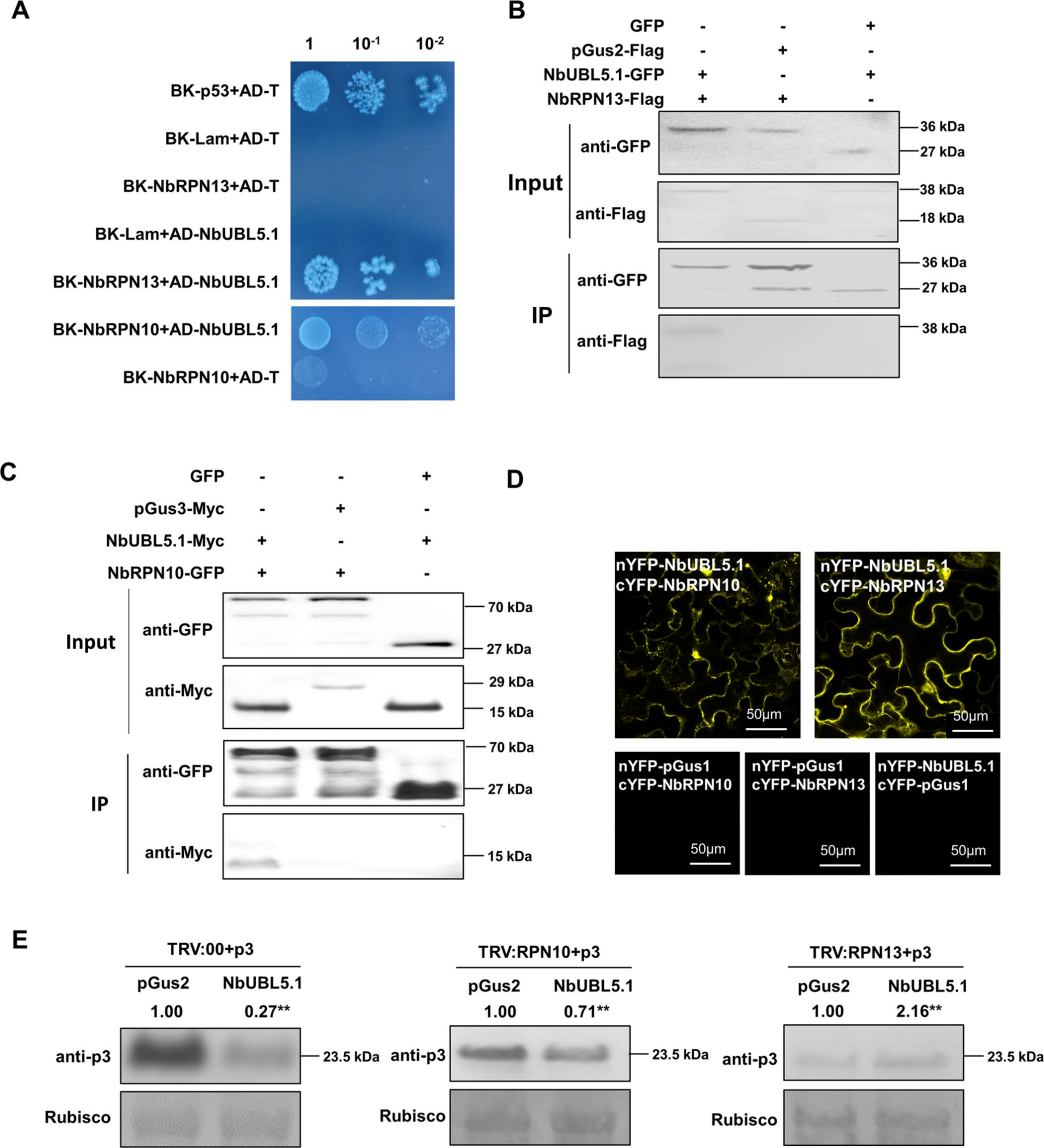
NbUBL5.1 interacts with the ubiquitin receptors in the 26S proteasome, NbRPN10 and NbRPN13, and their silencing inhibits NbUBL5.1-mediated p3 degradation. A. Y2H showing the interaction of NbUBL5.1 with NbRPN10 and NbRPN13. B and C Co-IP showing the interaction of NbUBL5.1 with NbRPN10 and NbRPN13. pGus3 (The N-terminal peptide of Gus with 199 amino acids) was used as control. D. BiFC showing the interaction of NbUBL5.1 with NbRPN10 and NbRPN13. E. On *NbRPN10*- and *NbRPN13*-silenced plants, a reduction of p3-GFP protein caused by NbUBL5.1-Myc was detected. Results indicate that their silencing inhibits NbUBL5.1-mediated p3 degradation. On non-silenced plants, reduction of p3-GFP protein caused by NbUBL5.1-Myc was used as control. GFP and Myc-fused proteins were detected by western blot. Results are from three biological replicates. Band intensity in blots was calculated by ImageJ based on three replicates. A two-sample unequal variance directional t test was used to test the significance of the difference (**, *p* value<0.01).

The 26S proteasome contains two subcomplexes, the core particle (CP, 20S) and the regulatory particle (RP, 19S). RPN10 and RPN13 are two subunits of the RP and function as ubiquitin receptors, delivering ubiquitinated proteins to the proteasome [39]. Interaction assays indicated that NbUBL5.1 interacted with both NbRPN10 and NbRPN13, suggesting the possibility that RPN10 and/or RPN13 recognize proteins interacting with UBL5 for delivery to the proteasome (Fig 5A–5D).

We next silenced either RPN10 or RPN13 and determined the NbUBL5.1-induced p3 reduction on the silenced plants. There was no severe phenotype as a result of silencing either *NbRPN10* or *NbRPN13* (S11A–S11C Fig). On plants where *NbRPN10* was silenced, reduction of p3 protein was alleviated when NbUBL5.1-Myc was co-expressed, compared to that on non-silenced plants (Fig 5E). In plants where *NbRPN13* was silenced, reduction of p3 protein was nearly completely inhibited (Fig 5E). The results suggest that the degradation of p3 mediated by NbUBL5 is RPN10/RPN13-dependent and it seems likely that NbRPN13 plays a main role in this process.

### OsUBL5.1 mediates p3 degradation through the 26S proteasome system and interacts with OsRPN10 and OsRPN13 from *O*. *sativa*

**Fig 6:**
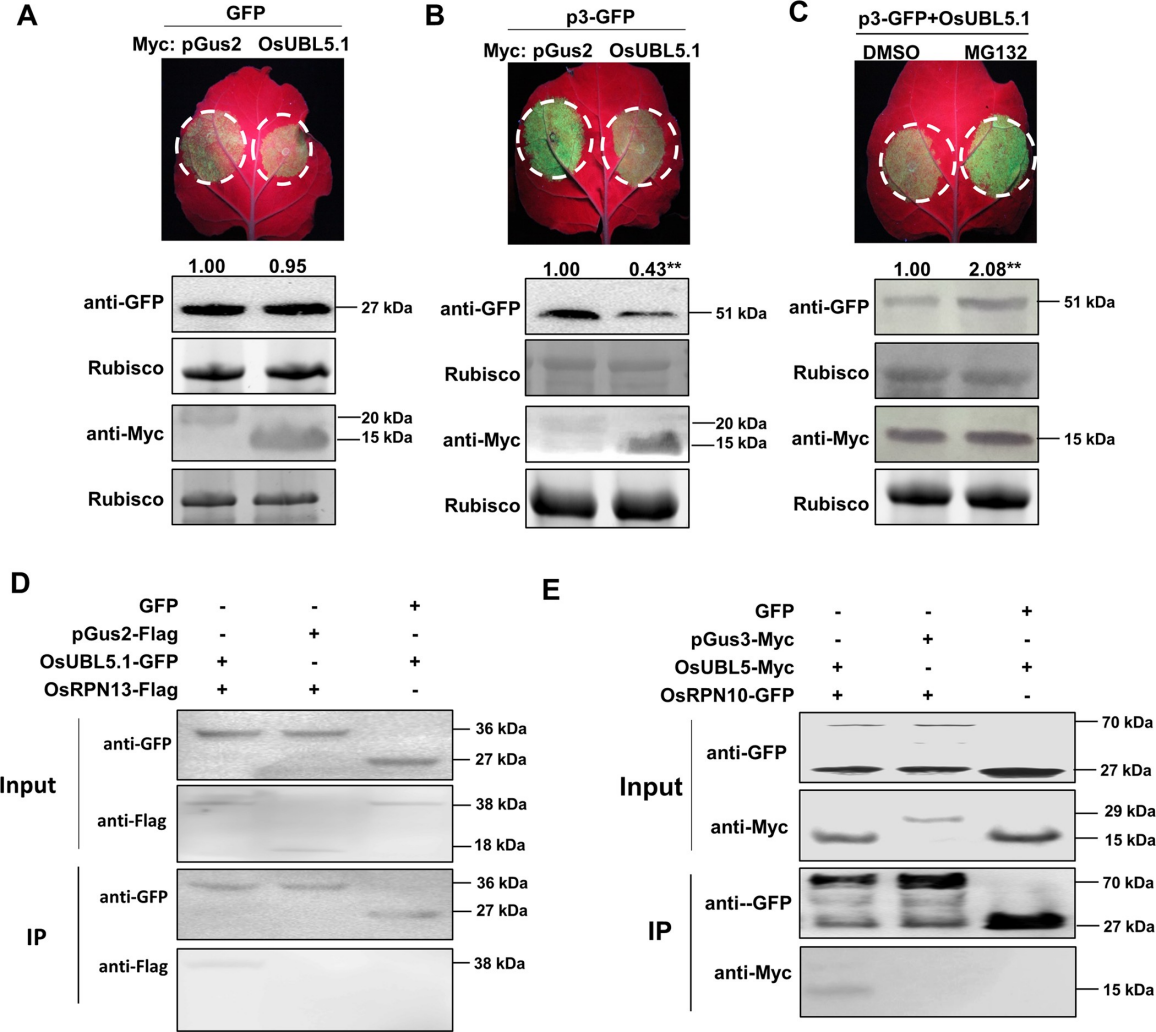
OsUBL5.1 mediates p3 degradation through tbe 26S proteasome system and interacts with OsRPN10 and OsRPN13 from *O*. *sativa*. A. Myc-fused OsUBL5 had no effect on GFP expression on leaves when it was co-expressed with GFP by agroinfiltration. B. Myc-fused OsUBL5 reduced GFP-fused p3 protein accumulation when they were co-expressed. C. Reduction of GFP-fused p3 protein caused by Myc-fused OsUBL5 was inhibited by MG132 treatment. Myc-fused pGus2 was used as control. GFP and Myc-fused proteins were detected by western blot. D and E. Co-IP showed the interaction of OsUBL5 with OsRPN13 and OsRPN10, respectively. Myc-fused pGus3 was used as control. Results are from three biological replicates. Band intensity in blots was calculated by ImageJ based on three replicates. A two-sample unequal variance directional t test was used to test the significance of the difference (**, *p* value<0.01).

OsUBL5.1 has high identity to NbUBL5s, and also interacts with p3. The ability of OsUBL5.1 to degrade p3 was therefore investigated. In the control, expression of Myc-fused OsUBL5.1 did not cause degradation of GFP, while the accumulation of p3-GFP was reduced when co-expressed with OsUBL5.1-Myc, similar to results from NbUBL5.1 (Fig 6A and 6B). Transcript levels of p3 were not affected in these experiments (S8C Fig). Moreover, this reduction of p3-GFP was inhibited by treatment with MG132 (Fig 6C, S12A and S12B Fig). OsUBL5.1 also interacted with OsRPN10 and OsRPN13 (Fig 6D and 6E, S13 Fig). Thus OsUBL5.1 interacts with p3 and mediates its degradation through the 26S proteasome system, indicating that it plays similar roles in rice to those of NbUBL5.1 in *N*. *benthamiana*.

### RSV infection is inhibited in plants overexpressing *UBL5*

**Fig 7:**
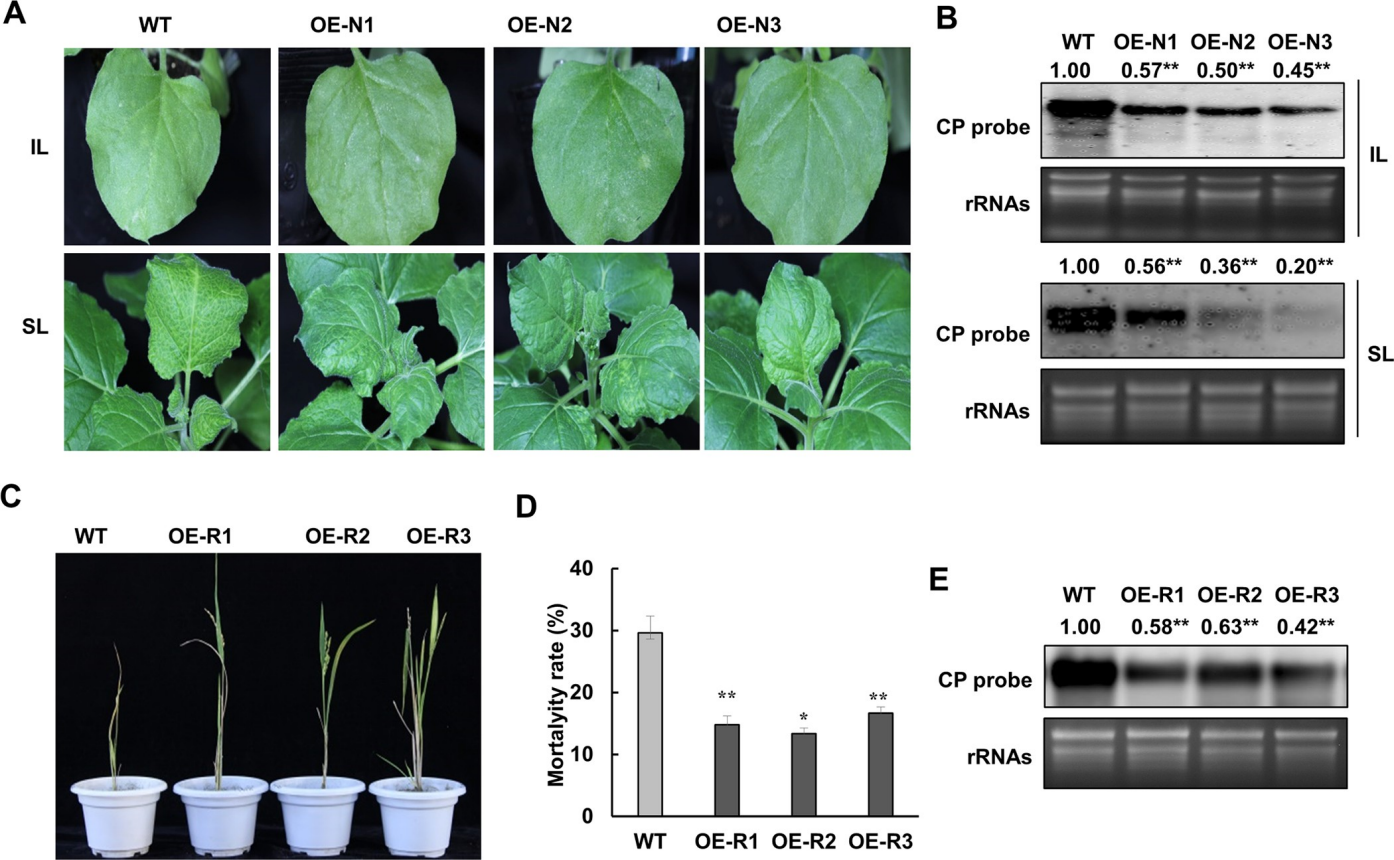
RSV infection is inhibited in plants overexpressing *UBL5*. A. *N*. *benthamiana* wild type and three transgenic lines (OE-N1, OE-N2 and OE-N3) overexpressing *NbUBL5*.*1* were inoculated with RSV. At 7 dpi, wild-type plants showed typical yellow foci on the inoculated leaves, while no obvious infection foci appeared on leaves of transgenic plants. At 15 dpi, all wild-type plants showed typical mosaic and chlorosis on newly-emerged leaves, implying systemic infection with RSV, but on transgenic plants the systemic symptom was significantly alleviated. B. Northern blot showing that RSV RNAs accumulated at a lower level in both the inoculated and systemically infected leaves of transgenic lines. C. Wild type rice and three transgenic lines (OE-R1, OE-R2 and OE-R3) overexpressing OsUBL5 were inoculated with RSV. At 20 dpi, approximately 40% of the wild type plants were dead, while only about 25% transgenic plants had died. D. In the surviving plants, RSV symptoms on transgenic plants were significantly milder with fewer yellow streaks than on wild type plants. E. In the surviving plants, the accumulation of RSV RNAs was also less than on transgenic plants. Twenty plants were used for each of three biological repeats. Bars represent the standard errors of the means from three biological repeats. Band intensity in blots was calculated by ImageJ based on three replicates. A two-sample unequal variance directional t test was used to test the significance of the difference (**, *p* value<0.01).

To further investigate the biological function of UBL5-mediated p3 degradation in RSV infection, we generated stable transgenic lines of *N*. *benthamiana* overexpressing cauliflower mosaic virus (CaMV) 35S-driven *NbUBL5*.*1* and studied RSV infection on the transgenic plants. Three independent lines (N1, N2 and N3) were identified with high levels of expression of *NbUBL5*.*1* by quantitative RT-PCR (S14A and S14B Fig). These transgenic plants had no obvious developmental phenotype. RSV was inoculated onto the transgenic plants and onto wild type plants as controls at the 5-leaf stage. At 7 dpi, wild-type plants showed typical yellow foci on the inoculated leaves, but there were no obvious infection foci on leaves of transgenic plants (Fig 7A). At 15 dpi, all wild-type plants showed typical mosaic and chlorosis on newly-emerged leaves, implying systemic infection with RSV, but the symptoms were significantly alleviated on transgenic plants (Fig 7A). In transgenic plants, RSV RNAs accumulated at a lower level in both the inoculated and systemically infected leaves (Fig 7B). Thus RSV infection was inhibited in *N*. *benthamiana* plants overexpressing *NbUBL5*.*1*. Moreover, when p3 was expressed transiently in transgenic plants, its accumulation was reduced compared to that in the wild type (S14C Fig).

We also created transgenic rice lines over-expressing *OsUBL5*.*1* for analysis. The full-length ORF of *OsUBL5*.*1* was overexpressed in plants under the control of the CaMV 35S promoter by agrobacterium-mediated transformation. Transgenic lines had no notable phenotypic differences from wild type rice plants in growth and development (S14D Fig). Three independent transgenic lines (R1, R2 and R3) with upregulated expression of *OsUBL5*.*1* were used for analysis (S14E Fig). At the three-leaf stage, plants were infested for three days with *L*. *striatellus* carrying RSV, and the subsequent viral infection on the rice seedlings was monitored. At 20 dpi, approximately 40% of the wild type plants were dead, while only about 15% of the transgenic plants had died (Fig 7C and 7D). In the surviving plants, RSV symptoms on transgenic plants were significantly milder with fewer yellow streaks than on wild type plants. RSV RNA accumulation was also less on transgenic plants (Fig 7E). Thus, overexpression of *OsUBL5*.*1* also inhibited RSV infection in rice.

## Discussion

**Fig 8:**
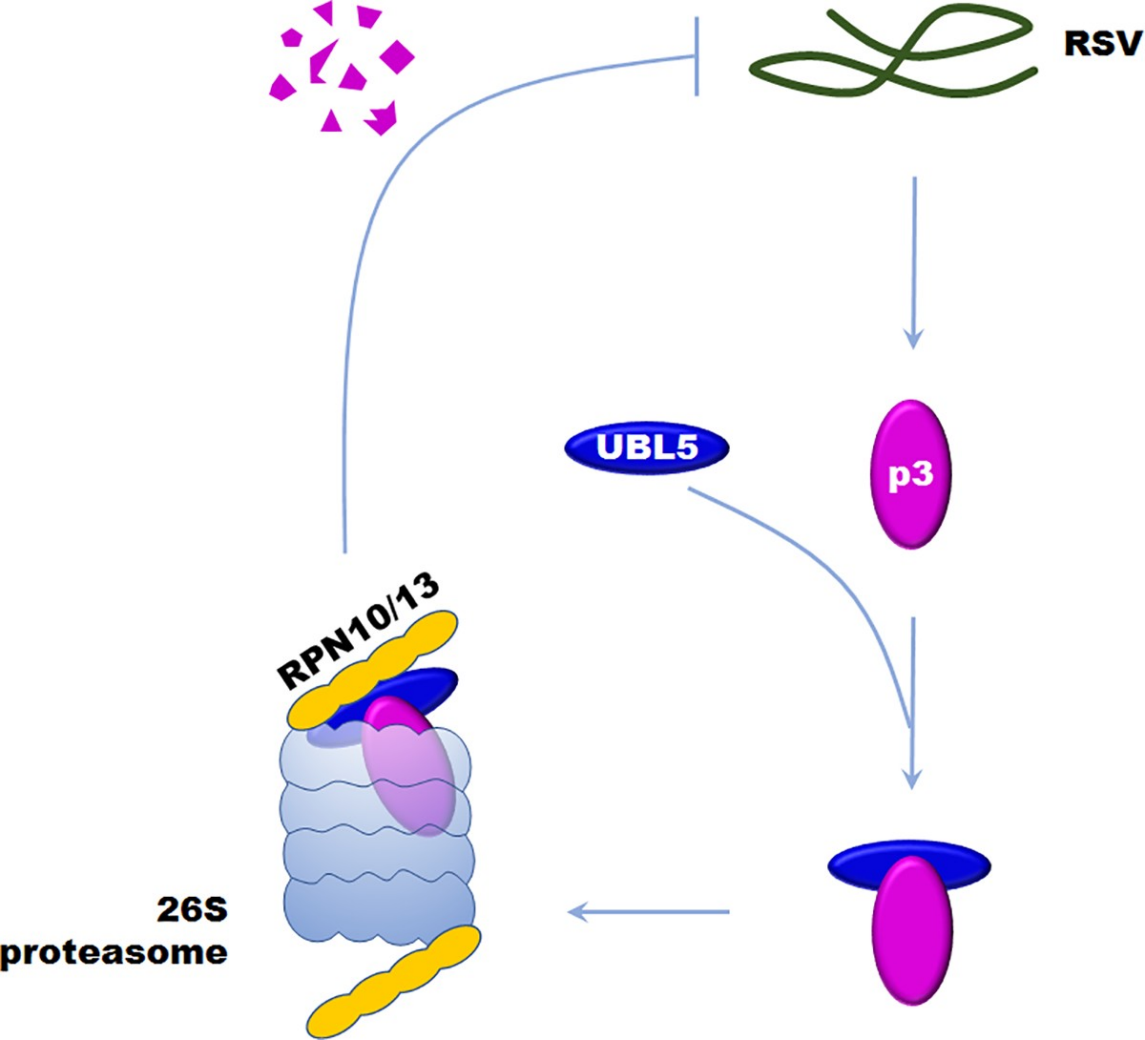
A possible working model of UBL5-mediated p3 degradation through the 26S proteasome as a defense against RSV infection. UBL5 interacts with p3 and is then recognized by RPN10 and RPN13 to be delivered into the 26S proteasome for p3 degradation, which inhibits the infection of RSV.

Ubiquitin is a covalent modifier of proteins that regulates the degradation of the modified proteins through the 26S proteasome system, which is conserved in higher eukaryotes. A large number of ubiquitin-like proteins (UBLs) have also been found in nearly all eukaryotic organisms [3] and these almost all contain a free di-glycine (GG) motif at the protruding carboxy-terminal end that is responsible for covalent conjugation to the target protein [21]. However, UBL5 lacks both the GG-motif and the exposed C-terminal tail, and also differs from ubiquitin because it conjugates with other proteins only in a non-covalent way [15, 16, 20, 21, 24, 25, 40]. Here, we found that UBL5 from either *N*. *benthamiana* or rice interacted with the p3 protein of RSV, and degraded it through the 26S proteasome (Fig 8). As far as we know, this is the first report that UBL5 interacts with a pathogen protein and that UBL5-mediated regulation might also participate in plant defense against pathogens [26, 27].

Previous reports indicate that UBL5 modulates the proteins with which it interacts by being part of a functional complex. For example, UBL5 interacts with Snu66 and helps it to play roles in pre-mRNA splicing at its correct subcellular location [20]. Many other UBLs function in a similar manner [41–45]. Small Ubiquitin-like Modifier (SUMO)-conjugated JAZ proteins bind to CORONATINE INSENSITIVE1 (COI1) to inhibit its function as a JA receptor [41]. SUMO also conjugates to FLS2, a receptor that perceives bacterial flagellin and PRR protein, triggering intracellular signaling in plant innate immunity[42]. SUMO3 ligates to replication protein NIb to facilitate turnip mosaic virus (TuMV) infection [43]. Our results suggest that UBL5 may also play a role in mediating target protein degradation, a previously unreported function and one that it shares with ubiquitin. The similarity in function is further shown because UBL5 interacted with RPN10 and RPN13, the major ubiquitin receptors in the 26S proteasome. Deletions of the ubiquitin receptors RPN10 and RPN13 are viable in yeast, indicating the redundancy of proteasomal Ub receptors [46, 47] but it is not known whether other proteasomal ubiquitin receptors also function in UBL5-mediated p3 degradation.

It has been reported that the HIND-binding surface (termed surface I) in yeast Hub1 binds to Snu66 and functions in the later stage of spliceosome assembly [21], while the opposite side of Hub1 (termed surface II) mediates Prp5 binding and functions during the early steps of spliceosome assembly [22, 23]. Here, mutation of two residues (N7 and D64) in surface II abolished the interaction of NbUBL5.1 with p3 (Fig 1), indicating that surface II in UBL5 mediates the interaction with RSV p3, although the fates of the proteins bound by this surface are different.

UBL5-mediated protein degradation is evidently specific because it is dependent on the interaction with UBL5. No plant proteins have yet been reported to be degraded by UBL5, and we therefore do not know what role such degradation may play in plant development or physiology. Meanwhile, silencing of *NbUBL5s* caused chlorosis on the top leaves of plants, indicating a potential function of *NbUBL5s* in chloroplast development (Fig 2). The results presented here demonstrate an essential role for UBL5s in plant defense against RSV infection and it is possible that UBL5-mediated protein degradation plays more important roles in plant immunity generally.

While the involvement of UBL5 in the 26S proteasome pathway is a novel finding, there have been increasing numbers of reports showing that the 26S proteasome plays important roles in virus-plant interactions by regulating or degrading viral components [48–51]. Polyubiquitination of tobacco mosaic virus (TMV) movement protein (MP) and subsequent degradation by the 26S proteasome regulates TMV systemic spread and reduces the damage caused by the MP on the structure of cortical ER [52]. The movement protein of turnip yellow mosaic virus (TYMV) is recognized as a substrate for the attachment of polyubiquitin chains and for subsequent rapid and selective proteolysis by the proteasome [53]. TYMV RdRp (termed 66K) is also a target of the ubiquitin-proteasome system in plant cells, by which viral replication is regulated [54]. The βC1 protein encoded by the TYLCCNV-associated betasatellite interacted with NtRFP1, a functional E3 ubiquitin ligase that mediates βC1 ubiquitination and degradation. Plants overexpressing NtRFP1 had attenuated symptoms [38]. Viruses also have multiple strategies to antagonize the host proteasome defense system. Potato virus Y (PVY) helper component-proteinase (HC-Pro) protein can interact with three Arabidopsis 20S proteasome subunits to interfere with the activity of the proteasome and thus enhance virus accumulation [55–57]. It has also been reported that the 26S proteasome plays a defensive role in protecting SBPH against RSV infection. The RSV p3 protein interacts with the RPN3 subunit of the 26S proteasome in SBPH and attenuates the host defense response by hijacking the 26S proteasome to facilitate viral replication [58]. We here demonstrate that the 26S proteasome functions in plants against RSV by targeting p3 for degradation, indicating that the interaction between the 26S proteasome and p3 differs between SBPH and plants (Fig 8). It has been reported that several viral proteins can inhibit the activity of the proteasome to enhance virus accumulation [55–57, 59]. We do not know whether RSV encodes viral proteins to inhibit the plant 26S proteasome during the molecular arms race between virus and plants, but this would be worth investigating next.

## Experimental procedures

### Plant growth and virus inoculation

Leaves infected with RSV were collected from a rice field in Jiaxing, Zhejiang Province, China, and stored at -80°C. *N*. *benthamiana* plants and transgenic plants overexpressing *NbUBL5*.*1* were grown in a greenhouse as previously reported [60]. *N*. *benthamiana* seeds were sown in pots with mixed soil matrix (peat: perlite: vermiculite = 3:1:3) and grown in a greenhouse with a 15-h light /9-h dark photoperiod at 24°C for 4–6 weeks. Five-week-old seedlings at the three leaf stage were selected to be infiltrated with *Agrobacterium tumefaciens* carrying TRV:00 or TRV:UBL5s. Real-time PCR was used to confirm the silencing of the *UBL5* gene and then RSV was mechanically inoculated to the middle leaves. During the period 8–17 days after inoculation, the number of infected plants was recorded every 8 hours. After RSV symptoms appeared on the top leaves, the leaves were photographed and collected for northern blot analysis. Rice (*O*. *sativa* spp. *japonica*) seedlings overexpressing *OsUBL5*.*1* were grown in clay loam soil in the greenhouse under 16 h light (30,000–60,000 lux)/8 h dark photoperiod at 28–30°C and 60 ± 5% relative humidity in a glass beaker. At the 3-leaf stage, SBPH was placed on the leaves and allowed to feed for 5 days. After removing the SBPH, plants were then transferred into field plots to grow until maturity and the incidence of infection was recorded over the following 30 days.

### Yeast two-hybrid assay

A rice cDNA library was constructed to screen for proteins interacting with RSVp3 using the GAL4 system as described in the BD Matchmaker Library Construction and Screening Kits User Manual (Clontech, Palo Alto, CA). RSV p3 was inserted into the yeast pGBKT7 vector and used as the bait by co-transformation into Y2H Gold yeast cells with the rice cDNA library. Transformants were grown on SD/-Leu/-Trp/-His medium, positive clones were selected on a histidine-deficient medium and then confirmed by X-α-Gal assays. To confirm the interaction between proteins, full length proteins were inserted into the pGADT7 vector (Clontech, Palo Alto, CA) as a prey. The corresponding proteins were introduced into pGBKT7 as a bait. Transformants were plated on synthetic defined solid medium lacking Leu and Trp (SD/-Leu/-Trp), grown for three days and then transferred onto synthetic defined medium lacking Ade, His, Leu and Trp with 70 μg ml^-1^ X-α–Gal (SD/-Ade/-His/-Leu/-Trp/X-α-Gal). pGBK-53 and pGAD-T were also co-transformed as a positive control, while pGBK-Lam and pGAD-T were co-transformed as a negative control. Three independent experiments were performed to confirm the results.

### BiFC

The full-length coding regions of *OsUBL5*.*1*, *OsUBL5*.*2*, *NbUBL5*.*1*, *OsRPN10*, *NbRPN10*, *OsRPN13* and *NbRPN13* were amplified from *O*. *sativa* spp. *japonica* and *N*. *benthamiana* cDNA using specific primers and fused either to the N-terminal or C-terminal fragment of yellow fluorescent protein (YFP). The plasmids were then individually transformed into *Agrobacterium tumefaciens* strain EHA105 by electroporation. Mixtures at equivalent concentrations were transiently co-expressed in fully expanded leaves of three-week-old *N*. *benthamiana*. The BiFC assays were performed as described previously and YFP fluorescent images were obtained using a confocal microscope (CLSM, Leica TCS SP5, Mannheim, Germany) at 48 dpi [61, 62].

### Co-IP assays

In assays to study the interaction between RSV p3 and either OsUBL5.1 or NbUBL5.1, GFP was fused with OsUBL5.1 and NbUBL5.1, producing OsUBL5.1-GFP and NbUBL5.1-GFP. p3 protein was fused to the N-terminus of the Myc label transient expression vector. Empty GFP and pGus2 were fused with Myc individually as negative controls. In assays to study the interaction between UBL5s (OsUBL5.1 or NbUBL5.1) and RPN proteins (OsRPN10, NbRPN10, OsRPN13, NbRPN13), the UBL5s were separately constructed into the Myc label expression vector, producing OsUBL5.1-Myc and NbUBL5.1-Myc while the RPN proteins were inserted into the N-terminus of the GFP label transient expression vector producing OsRPN10-GFP, NbRPN10-GFP, OsRPN13-GFP and NbRPN13-GFP.

After co-expressing the fusion proteins for 48 h in *N*. *benthamiana* cells, total proteins were extracted using GTEN buffer (10% glycerol, 25mM Tris-HCl pH 7.5, 1mM EDTA, 150mM NaCl), containing 10mM DTT, 1mM PMSF, 0.5% nonidet P40 and protease inhibitor cocktail. After standing on ice for 30 min to lyse the cells, samples were centrifuged twice at 13,200 rpm for 30 minutes, and the supernatant was transferred to the new Eppendorf tubes. Western blot was used to determine the expression levels of the proteins. 20 μL of GFP-Trap M beads (Chromo Tek, Martinsried, Germany) were added into total protein extracts and incubated for 2h. The beads were washed five times with 1.5 mL washing buffer, and then 4×protein loading buffer was added to the bound complexes and boiled for 10 min. Protein samples were separated by 12% SDS-PAGE gel electrophoresis and transferred onto nitrocellulose membrane (Amersham, Sweden) by semi-dry electroblotting. Labelled proteins were detected with anti-GFP or anti-Myc primary antibodies and an anti-mouse or anti-rabbit AP secondary antibody.

### VIGS

TRV vectors pTRV1 and pTRV2 were kindly provided by Dr. Yule Liu, Tsinghua University (Beijing, China) and used for VIGS [63]. A partial sequence from *NbUBL5*.*1*, *NbRPN10* and *NbRPN13* was amplified by specific primers and inserted into vector pTRV2 producing TRV:UBL5s, TRV:NbRPN10 and TRV:NbRPN13 (S1 Table). Empty vector, named TRV:00, was used for the control treatment. All plasmids were introduced into *Agrobacterium* strain EHA105 by electroporation (BIO-RAD, Hercules, CA, USA). The pTRV1 and TRV:UBL5s, TRV:NbRPN10 or TRV:NbRPN13 were mixed (1: 1 ratio) and infiltrated into *N*. *benthamiana* leaves at the four-leaf stage using a one-mL needleless syringe and the plants were then grown for 2 wk before analysis.

### Functional analysis of p3 as a suppressor of RNA silencing

The efficiency of p3 as a suppressor of RNA silencing was analyzed by the method previously described [31]. GFP, p3 and NbUBL5.1, OsUBL5.1 or pGus2 mixed cultures were co-expressed in 16c transgenic *N*. *benthamiana* leaves by agroinfiltration. The OD_600_ of *Agrobacterium tumefaciens* was 0.1. At 5 dpi, GFP fluorescence was excited using a hand-held 100-W, long-wave UV lamp (UV Products, Upland, CA, USA) and then photographed to analyze the intensity of fluorescence. Corresponding leaves were collected to analyze the accumulation of protein and RNAs. Experiments were repeated at least three times.

### Transient expression by agroinfiltration

Transient expression experiments were performed by *Agrobacterium tumefaciens*-mediated genetic transformation. Plasmids expressing the target genes were transformed into *A*. *tumefaciens* EHA105 strains and grown at 28°C for 48 h. After pelleting, re-suspending in infiltration buffer (10 mM MgCl_2_, 10 mM MES, and 200 μM acetosyringone, pH 5.6) and keeping at room temperature for 4 h, *A*. *tumefaciens* cultures (OD_600_ = 1.0) were infiltrated singly, or in 1:1 mixtures, into *N*. *benthamiana* leaves. At 3 to 6 dpi, infiltrated leaves were collected and stored at -80°C until assayed for expression levels.

### Western blot analysis

Infiltrated leaves were sampled using a hole punch and the pieces were then disrupted by oscillating in 90 μL protein lysis buffer (100 mM Tris-HCl, pH 8.8, 60% SDS, 2% β-mercaptoethanol) and standing on ice for 30 min. The crude extracts were centrifuged at 13,000 rpm for 20 minutes, the supernatants were transferred to 500 μL sterile tubes, mixed with 10 μL 10×SDS-PAGE loading buffer, boiled for 5 minutes and stood on ice for 2 minutes. Proteins were separated by electrophoresis on a 12% SDS-PAGE gel running at 80–130 V for 2 h and then transferred onto nitrocellulose membranes (Amersham, Uppsala, Sweden) by the semi-dry electroblotting method. The target protein was detected by a primary antibody against GFP or p3 diluted in 5% skim milk powder with TBS buffer for 2 h. The secondary antibody was a goat anti-mouse or anti-rabbit IgG conjugated with alkaline phosphatase (Sigma-Aldrich, St Louis, USA) or HRP anti-rabbit/mouse diluted to 1:10000 (v/v) in TBS buffer. Detection was visualized using nitrotetrazolium blue chloride/ 5-bromo-4-chloro-3-indolyl phosphate (NBT/BCIP) buffer (Sigma) under room conditions.

### RNA isolation and analysis

Total RNA was extracted from *N*. *benthamiana* leaf tissue by Trizol reagent according to the instruction manual (Invitrogen, Carlsbad, CA, USA). For real-time PCR analysis, 1μg RNA was used to synthesize first-strand cDNA as described in the reserve transcription reagent brochure (Takara, Shiga, Japan). Random primers or 21-nucleotide oligo (dT) were used for the experiment and at least three biological repeats were performed using three independent samples to ensure stabilized gene expression patterns. Three technical repeats of the reaction were performed on an Applied Biosystems ABI 7500 and at least three consistent data sets were used for analysis. Transcripts of the plant endogenous actin genes were used as reference controls. Quantitative results were calculated by the ΔΔC_T_ method. The sequences of primers used in the qPCR experiment are listed in supplemental S1 Table.

Mass molecular RNAs were separated by running 1.5% formaldehyde denaturing agarose gels and transferring to hybond-N^+^ membranes (Amersham Pharmacia, Piscataway, NJ, USA) for northern blot hybridization. 15 μg total RNAs were used for hybridization assays; equal volumes of RNAs and formamide deionized mixtures were denatured at 65°C for 10 min, the gel was run at 70 V for about 1h and samples were then transferred to membranes, thermofixed at 80°C for 2h, pre-hybridized at 37°C for 1 h and then hybridized overnight. The RSV *CP* full-length sequence was amplified from RSV-infected rice plants and four GFP fragments were amplified from a GFP expression vector using the primers listed in supplemental S1 Table. Probes were labelled with digoxin (DIG) according to the manufacturer’s protocol (DIG Oligonucleotide 3'-End Labeling Kit, Roche, Basel, Switzerland) and incubated at 37°C in a water bath for 16 h. Membranes were washed three times at 10 min intervals, incubated in blocking buffer for 45 minutes and then incubated with digoxigenin label AP secondary antibody for two hours. Hybridization signals were detected using CSPD (Vial 5) according to the protocol of the DIG High Prime DNA Labelling and Detection Starter Kit II (Roche) and band intensities were analyzed by ImageJ software (Wayne Rasband National Institutes of Health, Bethesda, MD, USA).

### MG132 treatment

Target proteins were co-expressed in *N*. *benthamiana* leaves by agroinfiltration for about 36 h, then 100 μM MG132 (or DMSO for the control) was injected into the treated leaves. After 20h, samples were punched from similarly-sized leaves to extract the total proteins by incubation in lysis buffer, standing on ice for 30 min and centrifugation at 13,200 rpm for 20 min. The supernatant was transferred into a new 1.5 mL Eppendorf tube, mixed with 10×SDS-PAGE loading buffer, boiled for 10 min, and then used for western blot analysis to detect the expression levels of the protein.

## Supporting information

S1 Fig Y2H (A) and BiFC (B) assays showing the interaction between OsUBL5.2 and RSV p3.
(TIF)

S2 Fig Three homologs of OsUBL5.1 in *N*. *benthamiana* were induced by RSV infection and interacted with p3 protein in Y2H assays. A. Amino acid sequences of three homologs of OsUBL5.1 in *N*. *benthamiana* Genome v1.0.1. The sites selected for analysis from among the conserved amino acids are indicated by triangles. B. The expression of *NbUBL5s* was up-regulated in RSV-infected *N*. *benthamiana* plants. Bars represent the standard errors of the means from three biological repeats. Two-sample unequal variance directional t test was used to test the significance of the difference (**, *p* value<0.01). C. BiFC showing the interaction of p3 with NbUBL5.1. D. Y2H assay showing the interaction of p3 with NbUBL5.2 and NbUBL5.3.
(TIF)

S3 Fig Interaction between NbUBL5.1 mutants and p3 as analyzed by BiFC. There was no interaction between the N7 or D64 mutants and p3. The partial GUS (pGus1) protein was used as a non-interacting, negative control. Bars, 50 μm.
(TIF)

S4 Fig Detection of TRV RNA accumulation in TRV:UBL5s-infected and TRV:00-infected plants. Northern blot analysis using a TRV CP probe showing that TRV RNAs in NbUBL5-silenced leaves (inoculated leaves and systemically infected leaves) accumulated at a similar level to the controls. Results are from three biological replicates. Band intensity in blots was calculated by ImageJ based on three replicates. A two-sample unequal variance directional t test was used to test the significance of the difference.
(TIF)

S5 Fig RSV infection on plants where *NbUBL5s* was silenced by the *NbUBL5*.*1* hairpin RNAi construct. **A.** Silencing of *NbUBL5s* by the hairpin RNAi construct (siUBL5s) did not cause obvious chlorosis on plants. B. Expression of *NbUBL5s* in *NbUBL5*.*1* hairpin RNAi construct-infiltrated plants decreased to 40% of the normal levels in control plants (siGus was infiltrated). C. Northern blot analysis showing that RSV RNAs in *NbUBL5s*-silenced leaves accumulated at higher levels than in controls. RSV CP probe was used for analysis. Band intensity in blots was calculated by ImageJ based on three replicates. A two-sample unequal variance directional t test was used to test the significance of the difference (**, *p* value<0.01).
(TIF)

S6 Fig Transient expression of *NbUBL5*.*1* had no effect on the endogenous RNA silencing pathway. A. Myc-fused p3 or control protein pGus2 were co-expressed with GFP in 16c leaves. At 5 dpi, there was strong fluorescence in zones expressing GFP and p3-Myc, but not in those expressing GFP and pGus2-Myc. GFP mRNA accumulated more in p3-Myc than in pGus2-Myc. Western blot showing the expression of Myc-fused pGus2 or p3. B. Myc-fused NbUBL5.1 or pGus2 were co-expressed with GFP in 16c leaves. At 5 dpi, there was no fluorescence in either treatment. Both GFP mRNAs and proteins in the zones expressing NbUBL5.1-Myc and GFP accumulated at similar levels to those in zones expressing pGus2-Myc and GFP. Results are from three biological replicates. Band intensity in blots was calculated by ImageJ based on three replicates. A two-sample unequal variance directional t test was used to test the significance of the difference (**, *p* value<0.01).
(TIF)

S7 Fig The VSR function of p19, HC-Pro and 2b was not affected by expression of NbUBL5.1-Myc. In the presence of NbUBL5.1-Myc, p19 (A), HC-Pro (B) and 2b (C) retained their VSR activity. Flag-fused VSRs were used for analysis. Results are from three biological replicates. Band intensity in blots was calculated by ImageJ based on three replicates. A two-sample unequal variance directional t test was used to test the significance of the difference.
(TIF)

S8 Fig Accumulation of p3 or p3-GFP transcripts as shown by northern blots. A. Accumulation of p3 transcripts when NbUBL5.1 or its mutant (D64A and R9A) was expressed in 16c. B. Accumulation of p3-GFP transcripts when NbUBL5.1 or its mutant (D64A and R9A) was expressed in wild type *N*. *benthamiana*. C. Accumulation of p3-GFP transcripts when OsUBL5.1 was expressed. Results are from three biological replicates. Band intensity in blots was calculated by ImageJ based on three replicates. A two-sample unequal variance directional t test was used to test the significance of the difference.
(TIF)

S9 Fig Accumulation of p3 in *NbUBL5s*-silenced leaves. p3-GFP was expressed in *NbUBL5s*-silenced leaves or non-silenced leaves by infiltration for 3 days, and then the samples were collected for analysis. The results show that p3-GFP protein accumulated more in silenced than in non-silenced leaves. Results are from three biological replicates. Band intensity in blots was calculated by ImageJ based on three replicates. A two-sample unequal variance directional t test was used to test the significance of the difference (**, *p* value<0.01).
(TIF)

S10 Fig Effect of MG132 treatment on accumulation of target proteins. A and B. MG132 treatment inhibited the degradation of tomato yellow leaf curl China virus (TYLCCNV) βC1 protein that was reported to be degraded through the 26S proteasome system (A), but had no effect on the accumulation of GFP (B), showing that MG132 treatment had worked. C. MG132 treatment had no effect on accumulation of NbUBL5.1. Results are from three biological replicates. Band intensity in blots was calculated by ImageJ based on three replicates. A two-sample unequal variance directional t test was used to test the significance of the difference (**, *p* value<0.01).
(TIF)

S11 Fig Silencing of *NbRPN10* and *NbRPN13* in *N*. *benthamiana*. A. Plant phenotype was not greatly affected by silencing of either *NbRPN10* or *NbRPN13*. B and C. qRT-PCR indicated the silencing of *NbRPN10* and *NbRPN13* in the treated plants. Results are from three biological replicates. Bars represent the standard errors of the means from three biological repeats. A two-sample unequal variance directional t test was used to test the significance of the difference (*, *p* value<0.05; **, *p* value<0.01).
(TIF)

S12 Fig Effect of MG132 treatment on accumulation of OsUBL5.1. MG132 treatment had no effect on accumulation of OsUBL5.1-GFP (A) or GFP in the presence of OsUBL5.1 (B). GFP and Myc-fused proteins were detected by western blot. Results are from three biological replicates. Band intensity in blots was calculated by ImageJ based on three replicates. A two-sample unequal variance directional t test was used to test the significance of the difference.
(TIF)

S13 Fig BiFC assay showing the interaction of OsUBL5.1 with OsRPN10 (A) and OsRPN13 (B).
(TIF)

S14 Fig Overexpression of *UBL5* in plants. A. The phenotypes of three independent lines (OE-N1, N2 and N3) overexpressing NbUBL5.1. B. qRT-PCR showing high levels of expression of *NbUBL5*.*1* in these lines. C. When p3 was expressed transiently in transgenic plants, its accumulation was reduced compared to that in wild type. D and E. Three independent lines (OE-R1, R2 and R3) overexpressing OsUBL5.1 were identified (D) showing high levels of expression of *OsUBL5s* by qRT-PCR (E). Bars represent the standard errors of the means from three biological repeats. Band intensity in blots was calculated by ImageJ based on three replicates. A two-sample unequal variance directional t test was used to test the significance of the difference (*, *p* value<0.05; **, *p* value<0.01).
(TIF)

S1 Table Primers used in experiments. (DOCX)

## References

[1] Clague MJ, Urbe S. Ubiquitin: same molecule, different degradation pathways. Cell. 2010;143(5):682–5. Epub 2010/11/30. 10.1016/j.cell.2010.11.012 . 21111229

[2] Mukhopadhyay D, Riezman H. Proteasome-independent functions of ubiquitin in endocytosis and signaling. Science. 2007;315(5809):201–5. Epub 2007/01/16. 10.1126/science.1127085 . 17218518

[3] Hochstrasser M. Origin and function of ubiquitin-like proteins. Nature. 2009;458(7237):422–9. Epub 2009/03/28. 10.1038/nature07958 19325621 PMC2819001

[4] Schwartz DC, Hochstrasser M. A superfamily of protein tags: ubiquitin, SUMO and related modifiers. Trends in biochemical sciences. 2003;28(6):321–8. Epub 2003/06/27. 10.1016/S0968-0004(03)00113-0 . 12826404

[5] Song L, Luo ZQ. Post-translational regulation of ubiquitin signaling. J Cell Biol. 2019;218(6):1776–86. Epub 2019/04/20. 10.1083/jcb.201902074 31000580 PMC6548142

[6] Streich FC Jr., Lima CD. Structural and functional insights to ubiquitin-like protein conjugation. Annu Rev Biophys. 2014;43:357–79. Epub 2014/04/30. 10.1146/annurev-biophys-051013-022958 24773014 PMC4118471

[7] Miura K, Hasegawa PM. Sumoylation and other ubiquitin-like post-translational modifications in plants. Trends Cell Biol. 2010;20(4):223–32. 10.1016/j.tcb.2010.01.007 WOS:000277235700006. 20189809

[8] Kerscher O, Felberbaum R, Hochstrasser M. Modification of proteins by ubiquitin and ubiquitin-like proteins. Annu Rev Cell Dev Biol. 2006;22:159–80. 10.1146/annurev.cellbio.22.010605.093503 . 16753028

[9] Vierstra RD. The expanding universe of ubiquitin and ubiquitin-like modifiers. Plant physiology. 2012;160(1):2–14. Epub 2012/06/14. 10.1104/pp.112.200667 22693286 PMC3440198

[10] A Maxwell Burroughs1 LMI, and L Aravind2. The natural history of ubiquitin and ubiquitin-related domains. 2012. 10.2741/3996 PMC5881585 22201813

[11] Downes B, Vierstra RD. Post-translational regulation in plants employing a diverse set of polypeptide tags. Biochem Soc Trans. 2005;33(Pt 2):393–9. 10.1042/BST0330393 . 15787614

[12] Flotho A, Melchior F. Sumoylation: a regulatory protein modification in health and disease. Annu Rev Biochem. 2013;82:357–85. 10.1146/annurev-biochem-061909-093311 . 23746258

[13] Radoslav I. Enchev1 BASaMP. Protein neddylation: beyond cullin-RING ligases. 2015 10.1038/nrm3919 25531226 PMC5131867

[14] Villarroya-Beltri C, Guerra S, Sanchez-Madrid F. ISGylation-a key to lock the cell gates for preventing the spread of threats. J Cell Sci. 2017;130(18):2961–9. 10.1242/jcs.205468 28842471

[15] Luders J, Pyrowolakis G, Jentsch S. The ubiquitin-like protein HUB1 forms SDS-resistant complexes with cellular proteins in the absence of ATP. EMBO reports. 2003;4(12):1169–74. Epub 2003/11/11. 10.1038/sj.embor.7400025 14608371 PMC1326417

[16] Yashiroda H, Tanaka K. Hub1 is an essential ubiquitin-like protein without functioning as a typical modifier in fission yeast. Genes to cells: devoted to molecular & cellular mechanisms. 2004;9(12):1189–97. Epub 2004/12/01. 10.1111/j.1365-2443.2004.00807.x . 15569151

[17] McNally T, Huang Q, Janis RS, Liu Z, Olejniczak ET, Reilly RM. Structural analysis of UBL5, a novel ubiquitin-like modifier. Protein science: a publication of the Protein Society. 2003;12(7):1562–6. Epub 2003/06/26. 10.1110/ps.0382803 12824502 PMC2323916

[18] Ramelot TA, Cort JR, Yee AA, Semesi A, Edwards AM, Arrowsmith CH, et al Solution structure of the yeast ubiquitin-like modifier protein Hub1. Journal of structural and functional genomics. 2003;4(1):25–30. Epub 2003/08/29. 10.1023/a:1024674220425 . 12943364

[19] Dittmar GA, Wilkinson CR, Jedrzejewski PT, Finley D. Role of a ubiquitin-like modification in polarized morphogenesis. Science. 2002;295(5564):2442–6. Epub 2002/03/30. 10.1126/science.1069989 . 11923536

[20] Wilkinson CR, Dittmar GA, Ohi MD, Uetz P, Jones N, Finley D. Ubiquitin-like protein Hub1 is required for pre-mRNA splicing and localization of an essential splicing factor in fission yeast. Current biology: CB. 2004;14(24):2283–8. Epub 2004/12/29. 10.1016/j.cub.2004.11.058 . 15620657

[21] Mishra SK, Ammon T, Popowicz GM, Krajewski M, Nagel RJ, Ares M Jr, et al Role of the ubiquitin-like protein Hub1 in splice-site usage and alternative splicing. Nature. 2011;474(7350):173–8. Epub 2011/05/27. 10.1038/nature10143 21614000 PMC3587138

[22] Karaduman R, Chanarat S, Pfander B, Jentsch S. Error-Prone Splicing Controlled by the Ubiquitin Relative Hub1. Molecular cell. 2017;67(3):423–32 e4. Epub 2017/07/18. 10.1016/j.molcel.2017.06.021 . 28712727

[23] Chanarat S, Mishra SK. Emerging Roles of Ubiquitin-like Proteins in Pre-mRNA Splicing. Trends in biochemical sciences. 2018;43(11):896–907. Epub 2018/10/03. 10.1016/j.tibs.2018.09.001 . 30269981

[24] Oka Y, Varmark H, Vitting-Seerup K, Beli P, Waage J, Hakobyan A, et al UBL5 is essential for pre-mRNA splicing and sister chromatid cohesion in human cells. EMBO reports. 2014;15(9):956–64. Epub 2014/08/06. 10.15252/embr.201438679 25092792 PMC4198039

[25] Ammon T, Mishra SK, Kowalska K, Popowicz GM, Holak TA, Jentsch S. The conserved ubiquitin-like protein Hub1 plays a critical role in splicing in human cells. Journal of molecular cell biology. 2014;6(4):312–23. Epub 2014/05/30. 10.1093/jmcb/mju026 24872507 PMC4141198

[26] Oka Y, Bekker-Jensen S, Mailand N. Ubiquitin-like protein UBL5 promotes the functional integrity of the Fanconi anemia pathway. The EMBO journal. 2015;34(10):1385–98. Epub 2015/04/12. 10.15252/embj.201490376 25862789 PMC4491998

[27] Hatanaka K, Ikegami K, Takagi H, Setou M. Hypo-osmotic shock induces nuclear export and proteasome-dependent decrease of UBL5. Biochemical and biophysical research communications. 2006;350(3):610–5. Epub 2006/10/10. 10.1016/j.bbrc.2006.09.093 . 17026961

[28] Patel M, Milla-Lewis S, Zhang W, Templeton K, Reynolds WC, Richardson K, et al Overexpression of ubiquitin-like LpHUB1 gene confers drought tolerance in perennial ryegrass. Plant biotechnology journal. 2015;13(5):689–99. Epub 2014/12/10. 10.1111/pbi.12291 . 25487628

[29] Zhang C, Pei X, Wang Z, Jia S, Guo S, Zhang Y, et al The Rice stripe virus pc4 functions in movement and foliar necrosis expression in Nicotiana benthamiana. Virology. 2012;425(2):113–21. 10.1016/j.virol.2012.01.007 . 22305130

[30] Xiong R, Wu J, Zhou Y, Zhou X. Identification of a movement protein of the tenuivirus rice stripe virus. Journal of virology. 2008;82(24):12304–11. 10.1128/JVI.01696-08 . 18818319 PMC2593352

[31] Xiong R, Wu J, Zhou Y, Zhou X. Characterization and subcellular localization of an RNA silencing suppressor encoded by Rice stripe tenuivirus. Virology. 2009;387(1):29–40. 10.1016/j.virol.2009.01.045 . 19251298

[32] Shen M, Xu Y, Jia R, Zhou X, Ye K. Size-independent and noncooperative recognition of dsRNA by the Rice stripe virus RNA silencing suppressor NS3. Journal of molecular biology. 2010;404(4):665–79. 10.1016/j.jmb.2010.10.007 . 20951141

[33] Kim H, Cho WK, Lian S, Kim KH. Identification of residues or motif(s) of the rice stripe virus NS3 protein required for self-interaction and for silencing suppressor activity. Virus research. 2017;235:14–23. Epub 2017/04/11. 10.1016/j.virusres.2017.03.022 . 28392445

[34] Wu G, Zheng G, Hu Q, Ma M, Li M, Sun X, et al NS3 Protein from Rice stripe virus affects the expression of endogenous genes in Nicotiana benthamiana. Virology journal. 2018;15(1):105 Epub 2018/06/27. 10.1186/s12985-018-1014-7 29940994 PMC6019303

[35] Wu G, Wang J, Yang Y, Dong B, Wang Y, Sun G, et al Transgenic rice expressing rice stripe virus NS3 protein, a suppressor of RNA silencing, shows resistance to rice blast disease. Virus genes. 2014;48(3):566–9. Epub 2014/02/22. 10.1007/s11262-014-1051-2 . WOS:000336399600022. 24557730

[36] Zheng L, Zhang C, Shi C, Yang Z, Wang Y, Zhou T, et al Rice stripe virus NS3 protein regulates primary miRNA processing through association with the miRNA biogenesis factor OsDRB1 and facilitates virus infection in rice. PLoS pathogens. 2017;13(10):e1006662 Epub 2017/10/05. 10.1371/journal.ppat.1006662 28977024 PMC5658190

[37] Du Y, Zhao J, Chen T, Liu Q, Zhang H, Wang Y, et al Type I J-domain NbMIP1 proteins are required for both Tobacco mosaic virus infection and plant innate immunity. PLoS pathogens. 2013;9(10):e1003659 Epub 2013/10/08. 10.1371/journal.ppat.1003659 24098120 PMC3789785

[38] Shen Q, Hu T, Bao M, Cao L, Zhang H, Song F, et al Tobacco RING E3 Ligase NtRFP1 Mediates Ubiquitination and Proteasomal Degradation of a Geminivirus-Encoded betaC1. Molecular plant. 2016;9(6):911–25. Epub 2016/03/29. 10.1016/j.molp.2016.03.008 . 27018391

[39] Hamazaki J, Hirayama S, Murata S. Redundant Roles of Rpn10 and Rpn13 in Recognition of Ubiquitinated Proteins and Cellular Homeostasis. PLoS genetics. 2015;11(7):e1005401 Epub 2015/07/30. 10.1371/journal.pgen.1005401 26222436 PMC4519129

[40] Kantham L, Kerr-Bayles L, Godde N, Quick M, Webb R, Sunderland T, et al Beacon interacts with cdc2/cdc28-like kinases. Biochemical and biophysical research communications. 2003;304(1):125–9. Epub 2003/04/23. 10.1016/s0006-291x(03)00549-7 . 12705895

[41] Srivastava AK, Orosa B, Singh P, Cummins I, Walsh C, Zhang C, et al SUMO Suppresses the Activity of the Jasmonic Acid Receptor CORONATINE INSENSITIVE1. Plant Cell. 2018;30(9):2099–115. 10.1105/tpc.18.00036 30115737 PMC6181023

[42] Orosa B, Yates G, Verma V, Srivastava AK, Srivastava M, Campanaro A, et al SUMO conjugation to the pattern recognition receptor FLS2 triggers intracellular signalling in plant innate immunity. Nat Commun. 2018;9(1):5185 10.1038/s41467-018-07696-8 30518761 PMC6281677

[43] Cheng X, Xiong R, Li Y, Li F, Zhou X, Wang A. Sumoylation of Turnip mosaic virus RNA Polymerase Promotes Viral Infection by Counteracting the Host NPR1-Mediated Immune Response. The Plant cell. 2017;29(3):508–25. Epub 2017/02/23. 10.1105/tpc.16.00774 28223439 PMC5385955

[44] Ross S, Best JL, Zon LI, Gill G. SUMO-1 modification represses Sp3 transcriptional activation and modulates its subnuclear localization. Mol Cell. 2002;10(4):831–42. 10.1016/s1097-2765(02)00682-2 . 12419227

[45] Su CI, Tseng CH, Yu CY, Lai MMC. SUMO Modification Stabilizes Dengue Virus Nonstructural Protein 5 To Support Virus Replication. J Virol. 2016;90(9):4308–19. 10.1128/JVI.00223-16 26889037 PMC4836324

[46] Schreiner P, Chen X, Husnjak K, Randles L, Zhang N, Elsasser S, et al Ubiquitin docking at the proteasome through a novel pleckstrin-homology domain interaction. Nature. 2008;453(7194):548–52. Epub 2008/05/24. 10.1038/nature06924 18497827 PMC2825158

[47] Husnjak K, Elsasser S, Zhang N, Chen X, Randles L, Shi Y, et al Proteasome subunit Rpn13 is a novel ubiquitin receptor. Nature. 2008;453(7194):481–8. Epub 2008/05/24. 10.1038/nature06926 18497817 PMC2839886

[48] Marino D, Peeters N, Rivas S. Ubiquitination during plant immune signaling. Plant physiology. 2012;160(1):15–27. Epub 2012/06/13. 10.1104/pp.112.199281 22689893 PMC3440193

[49] Alcaide-Loridan C, Jupin I. Ubiquitin and plant viruses, let's play together! Plant physiology. 2012;160(1):72–82. Epub 2012/07/18. 10.1104/pp.112.201905 22802610 PMC3440231

[50] Dielen AS, Badaoui S, Candresse T, German-Retana S. The ubiquitin/26S proteasome system in plant-pathogen interactions: a never-ending hide-and-seek game. Molecular plant pathology. 2010;11(2):293–308. Epub 2010/05/08. 10.1111/j.1364-3703.2009.00596.x 20447278 PMC6640532

[51] Verchot J. Plant Virus Infection and the Ubiquitin Proteasome Machinery: Arms Race along the Endoplasmic Reticulum. Viruses. 2016;8(11). Epub 2016/11/22. 10.3390/v8110314 27869775 PMC5127028

[52] Reichel C, Beachy RN. Degradation of tobacco mosaic virus movement protein by the 26S proteasome. Journal of virology. 2000;74(7):3330–7. Epub 2000/03/09. 10.1128/jvi.74.7.3330-3337.2000 10708450 PMC111834

[53] Drugeon G, Jupin I. Stability in vitro of the 69K movement protein of Turnip yellow mosaic virus is regulated by the ubiquitin-mediated proteasome pathway. The Journal of general virology. 2002;83(Pt 12):3187–97. Epub 2002/12/06. 10.1099/0022-1317-83-12-3187 . 12466497

[54] Camborde L, Planchais S, Tournier V, Jakubiec A, Drugeon G, Lacassagne E, et al The ubiquitin-proteasome system regulates the accumulation of Turnip yellow mosaic virus RNA-dependent RNA polymerase during viral infection. The Plant cell. 2010;22(9):3142–52. Epub 2010/09/09. 10.1105/tpc.109.072090 20823192 PMC2965540

[55] Jin Y, Ma D, Dong J, Jin J, Li D, Deng C, et al HC-Pro protein of Potato virus Y can interact with three Arabidopsis 20S proteasome subunits in planta. J Virol. 2007;81(23):12881–8. 10.1128/JVI.00913-07 17898064 PMC2169110

[56] Ballut L, Drucker M, Pugniere M, Cambon F, Blanc S, Roquet F, et al HcPro, a multifunctional protein encoded by a plant RNA virus, targets the 20S proteasome and affects its enzymic activities. J Gen Virol. 2005;86(Pt 9):2595–603. 10.1099/vir.0.81107-0 . 16099919

[57] Sahana N, Kaur H, Basavaraj, Tena F, Jain RK, Palukaitis P, et al Inhibition of the host proteasome facilitates papaya ringspot virus accumulation and proteosomal catalytic activity is modulated by viral factor HcPro. PloS one. 2012;7(12):e52546 Epub 2013/01/10. 10.1371/journal.pone.0052546 23300704 PMC3531422

[58] Xu Y, Wu J, Fu S, Li C, Zhu ZR, Zhou X. Rice Stripe Tenuivirus Nonstructural Protein 3 Hijacks the 26S Proteasome of the Small Brown Planthopper via Direct Interaction with Regulatory Particle Non-ATPase Subunit 3. Journal of virology. 2015;89(8):4296–310. Epub 2015/02/06. 10.1128/JVI.03055-14 . 25653432 PMC4442352

[59] Li Y, Sun Q, Zhao T, Xiang H, Zhang X, Wu Z, et al Interaction between Brassica yellows virus silencing suppressor P0 and plant SKP1 facilitates stability of P0 in vivo against degradation by proteasome and autophagy pathways. The New phytologist. 2019;222(3):1458–73. Epub 2019/01/22. 10.1111/nph.15702 30664234 PMC6593998

[60] Shi B, Lin L, Wang S, Guo Q, Zhou H, Rong L, et al Identification and regulation of host genes related to Rice stripe virus symptom production. The New phytologist. 2016;209(3):1106–19. Epub 2015/10/22. 10.1111/nph.13699 . 26487490

[61] Jiang S, Lu Y, Li K, Lin L, Zheng H, Yan F, et al Heat shock protein 70 is necessary for Rice stripe virus infection in plants. Molecular plant pathology. 2014;15(9):907–17. Epub 2014/05/16. 10.1111/mpp.12153 . 24823923 PMC6638618

[62] Lu Y, Yan F, Guo W, Zheng H, Lin L, Peng J, et al Garlic virus X 11-kDa protein granules move within the cytoplasm and traffic a host protein normally found in the nucleolus. Molecular plant pathology. 2011;12(7):666–76. Epub 2011/07/06. 10.1111/j.1364-3703.2010.00699.x . 21726366 PMC6640471

[63] Liu Y, Schiff M, Marathe R, Dinesh-Kumar SP. Tobacco Rar1, EDS1 and NPR1/NIM1 like genes are required for N-mediated resistance to tobacco mosaic virus. Plant J. 2002;30(4):415–29. 10.1046/j.1365-313x.2002.01297.x . 12028572

