# Supplementary figures and images for: Ubiquitin-Like protein 5 interacts with the silencing suppressor p3 of rice stripe virus and mediates its degradation through the 26S proteasome pathway
Source: PLoS Pathog. 2020 Aug 31;16(8):e1008780. doi: 10.1371/journal.ppat.1008780 (PMC7485977; doi:10.1371/journal.ppat.1008780)

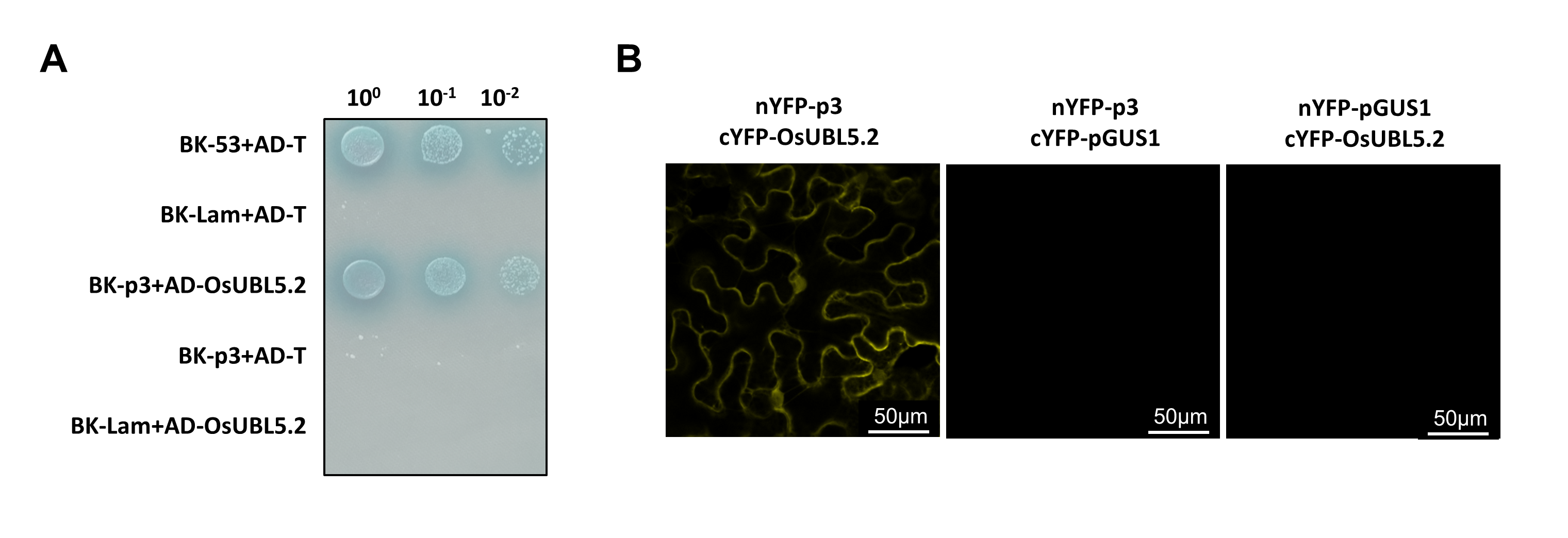

Supplement: S1 Fig — Y2H (A) and BiFC (B) assays showing the interaction between OsUBL5.2 and RSV p3. (TIF) [file ppat.1008780.s001.TIF]

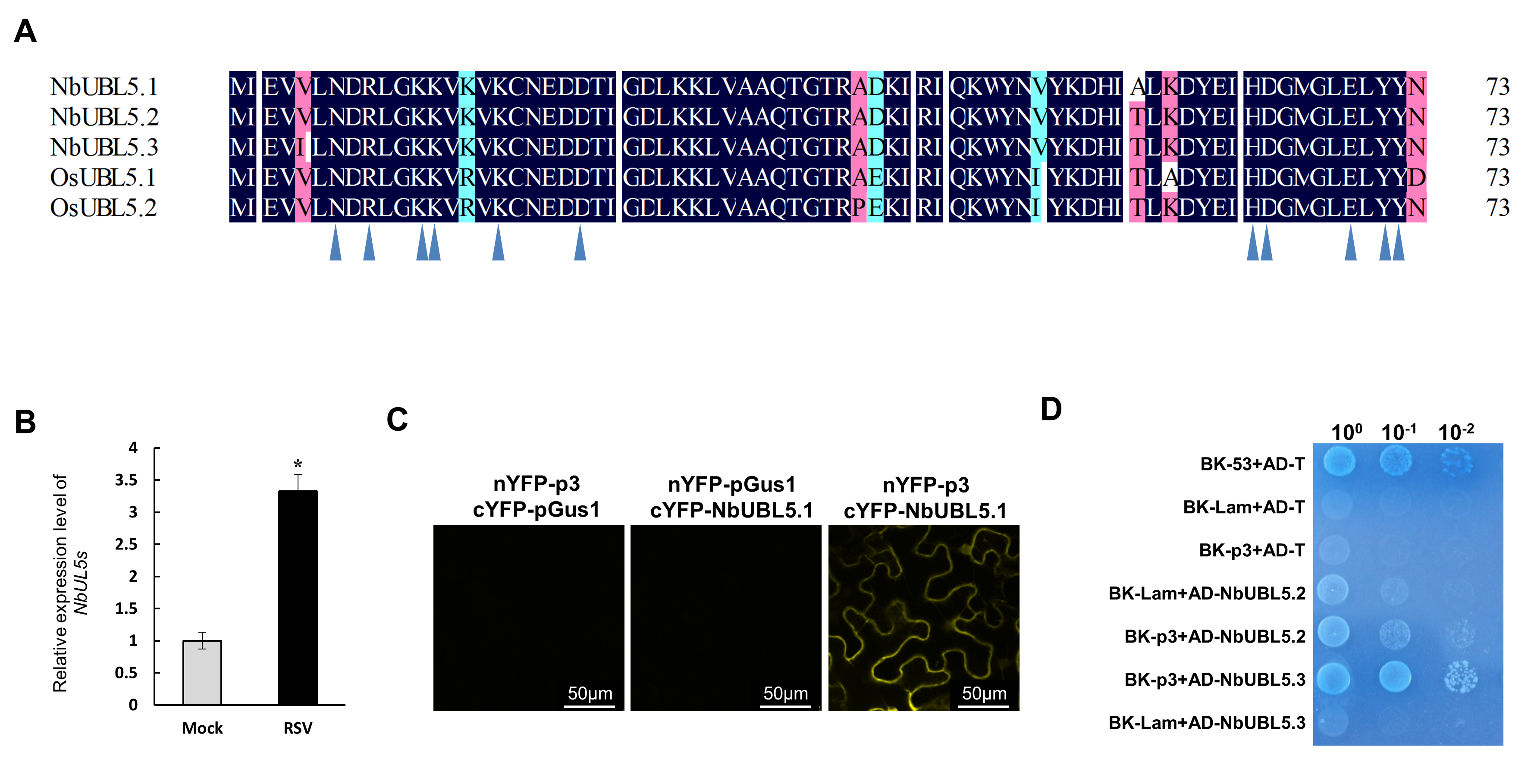

Supplement: S2 Fig — A. Amino acid sequences of three homologs of OsUBL5.1 in N. benthamiana Genome v1.0.1. The sites selected for analysis from among the conserved amino acids are indicated by triangles. B. The expression of NbUBL5s was up-regulated in RSV-infected N. benthamiana plants. Bars represent the standard errors of the means from three biological repeats. Two-sample unequal variance directional t test was used to test the significance of the difference (**, p value<0.01). C. BiFC showing the interaction of p3 with NbUBL5.1. D. Y2H assay showing the interaction of p3 with NbUBL5.2 and NbUBL5.3. (TIF) [file ppat.1008780.s002.TIF]

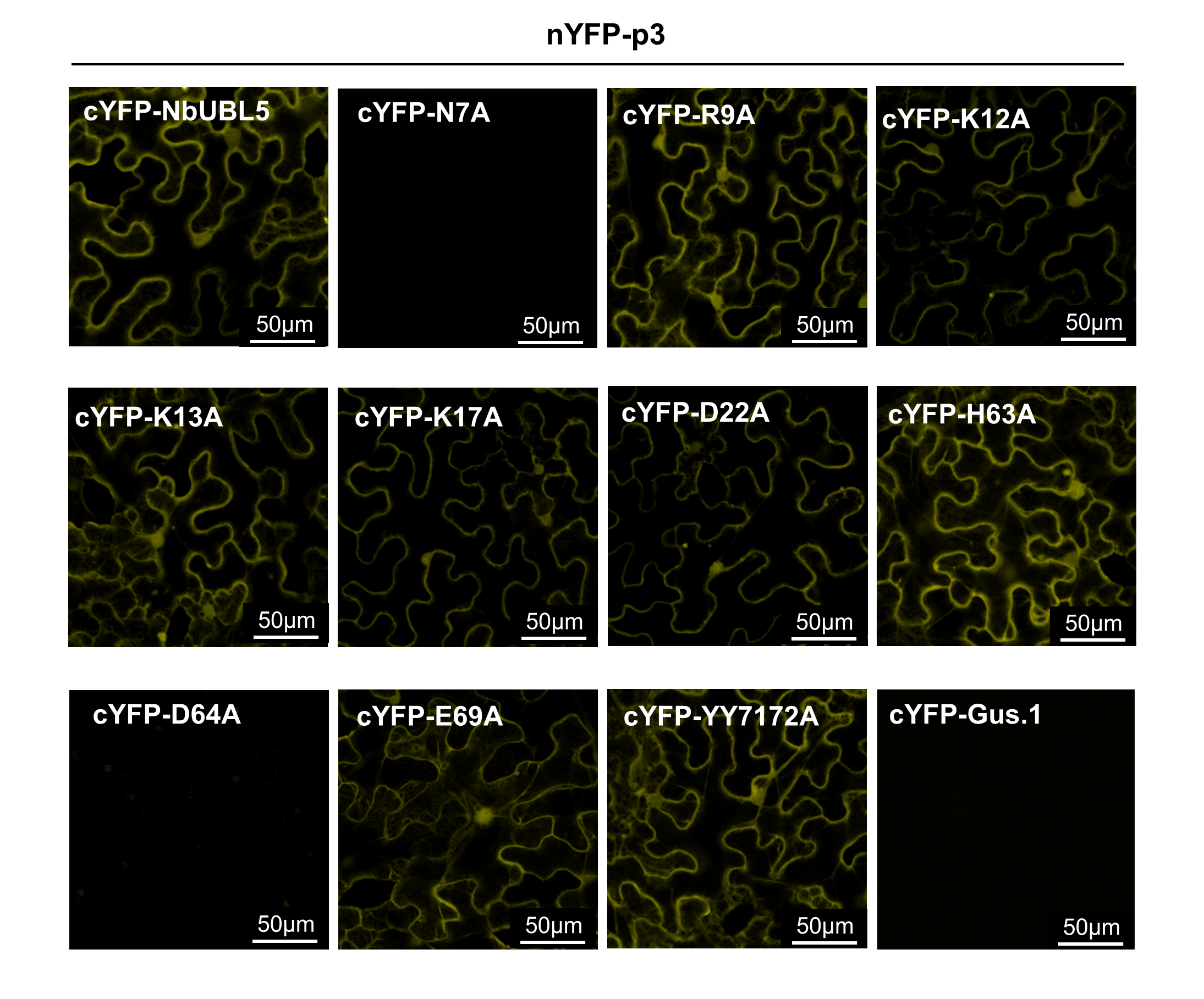

Supplement: S3 Fig — There was no interaction between the N7 or D64 mutants and p3. The partial GUS (pGus1) protein was used as a non-interacting, negative control. Bars, 50 μm. (TIF) [file ppat.1008780.s003.TIF]

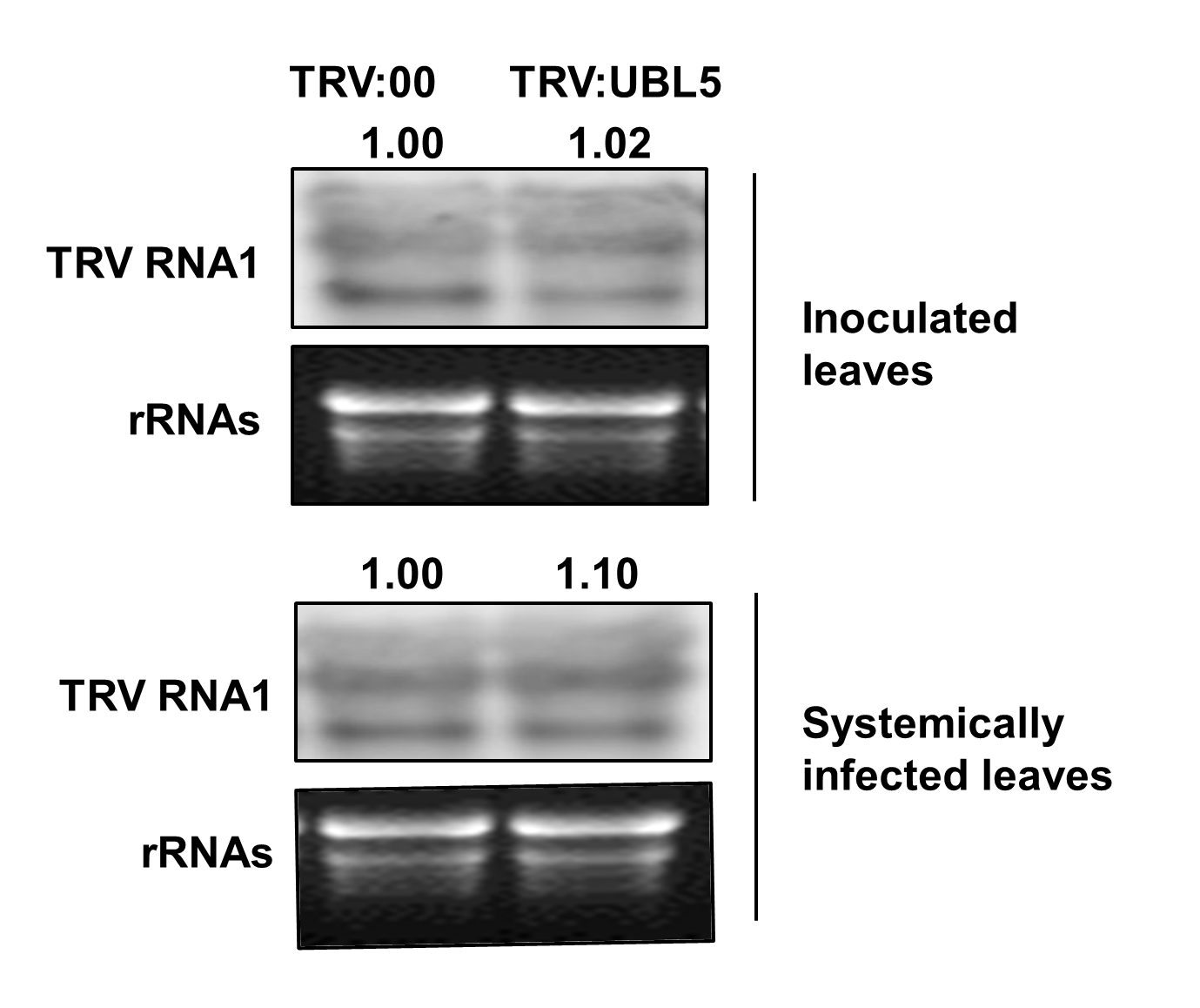

Supplement: S4 Fig — Northern blot analysis using a TRV CP probe showing that TRV RNAs in NbUBL5-silenced leaves (inoculated leaves and systemically infected leaves) accumulated at a similar level to the controls. Results are from three biological replicates. Band intensity in blots was calculated by ImageJ based on three replicates. A two-sample unequal variance directional t test was used to test the significance of the difference. (TIF) [file ppat.1008780.s004.TIF]

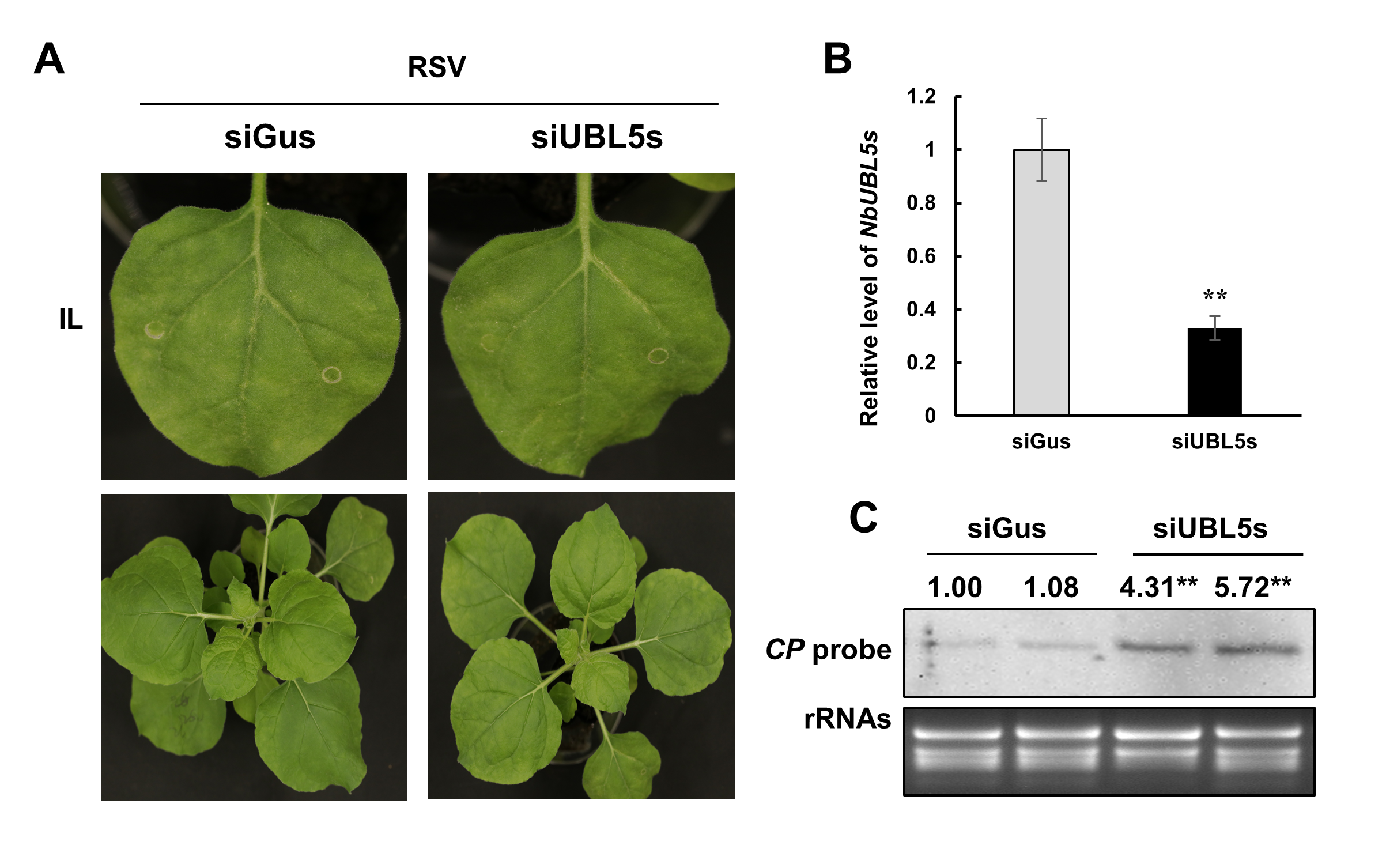

Supplement: S5 Fig — A. Silencing of NbUBL5s by the hairpin RNAi construct (siUBL5s) did not cause obvious chlorosis on plants. B. Expression of NbUBL5s in NbUBL5.1 hairpin RNAi construct-infiltrated plants decreased to 40% of the normal levels in control plants (siGus was infiltrated). C. Northern blot analysis showing that RSV RNAs in NbUBL5s-silenced leaves accumulated at higher levels than in controls. RSV CP probe was used for analysis. Band intensity in blots was calculated by ImageJ based on three replicates. A two-sample unequal variance directional t test was used to test the significance of the difference (**, p value<0.01). (TIF) [file ppat.1008780.s005.TIF]

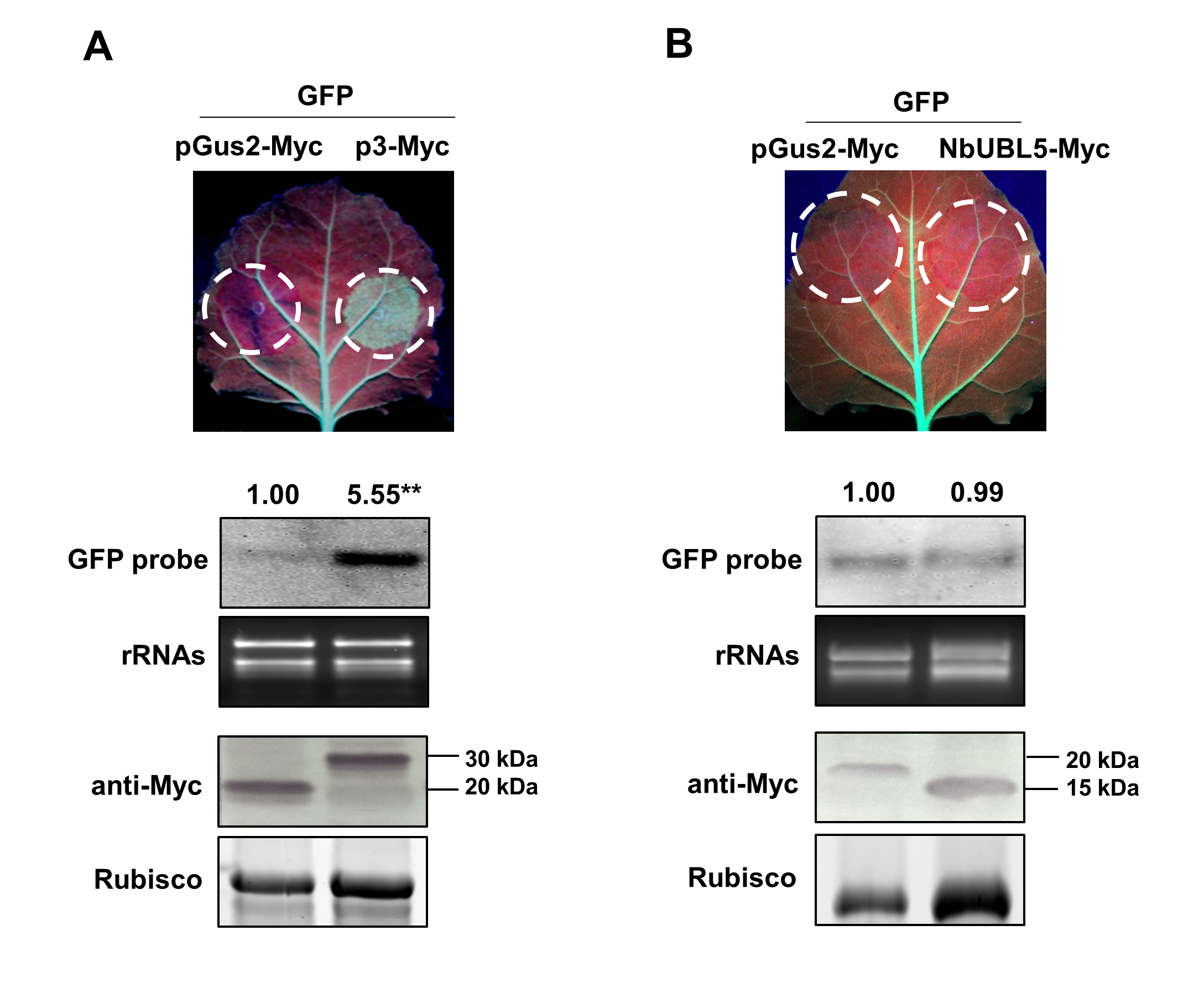

Supplement: S6 Fig — A. Myc-fused p3 or control protein pGus2 were co-expressed with GFP in 16c leaves. At 5 dpi, there was strong fluorescence in zones expressing GFP and p3-Myc, but not in those expressing GFP and pGus2-Myc. GFP mRNA accumulated more in p3-Myc than in pGus2-Myc. Western blot showing the expression of Myc-fused pGus2 or p3. B. Myc-fused NbUBL5.1 or pGus2 were co-expressed with GFP in 16c leaves. At 5 dpi, there was no fluorescence in either treatment. Both GFP mRNAs and proteins in the zones expressing NbUBL5.1-Myc and GFP accumulated at similar levels to those in zones expressing pGus2-Myc and GFP. Results are from three biological replicates. Band intensity in blots was calculated by ImageJ based on three replicates. A two-sample unequal variance directional t test was used to test the significance of the difference (**, p value<0.01). (TIF) [file ppat.1008780.s006.TIF]

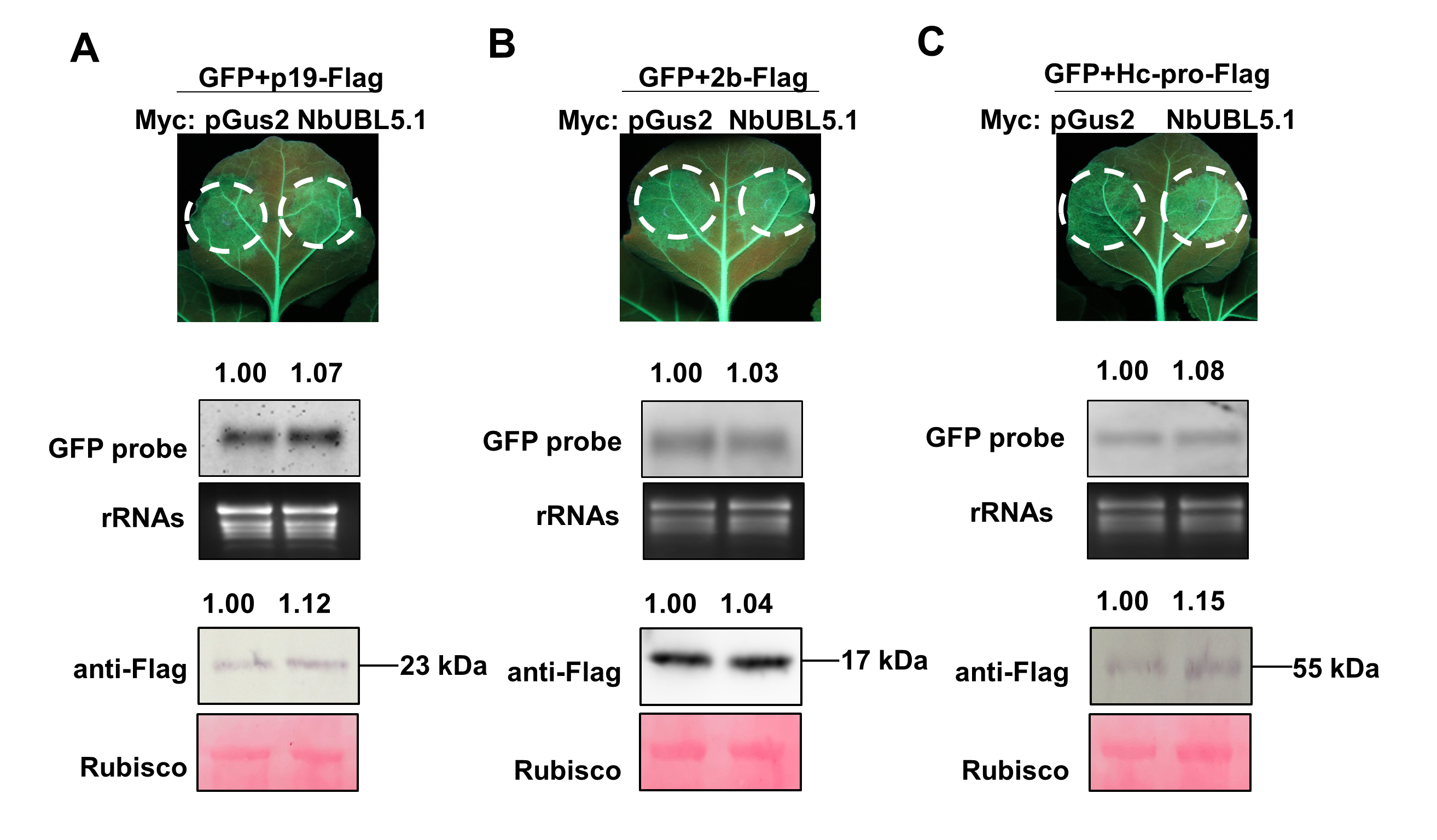

Supplement: S7 Fig — In the presence of NbUBL5.1-Myc, p19 (A), HC-Pro (B) and 2b (C) retained their VSR activity. Flag-fused VSRs were used for analysis. Results are from three biological replicates. Band intensity in blots was calculated by ImageJ based on three replicates. A two-sample unequal variance directional t test was used to test the significance of the difference. (TIF) [file ppat.1008780.s007.TIF]

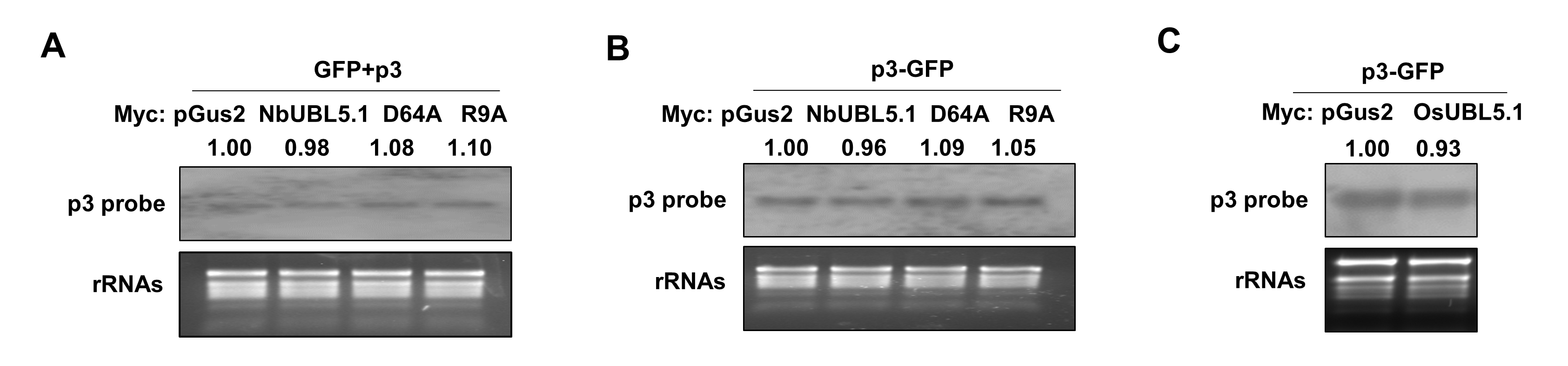

Supplement: S8 Fig — A. Accumulation of p3 transcripts when NbUBL5.1 or its mutant (D64A and R9A) was expressed in 16c. B. Accumulation of p3-GFP transcripts when NbUBL5.1 or its mutant (D64A and R9A) was expressed in wild type N. benthamiana. C. Accumulation of p3-GFP transcripts when OsUBL5.1 was expressed. Results are from three biological replicates. Band intensity in blots was calculated by ImageJ based on three replicates. A two-sample unequal variance directional t test was used to test the significance of the difference. (TIF) [file ppat.1008780.s008.TIF]

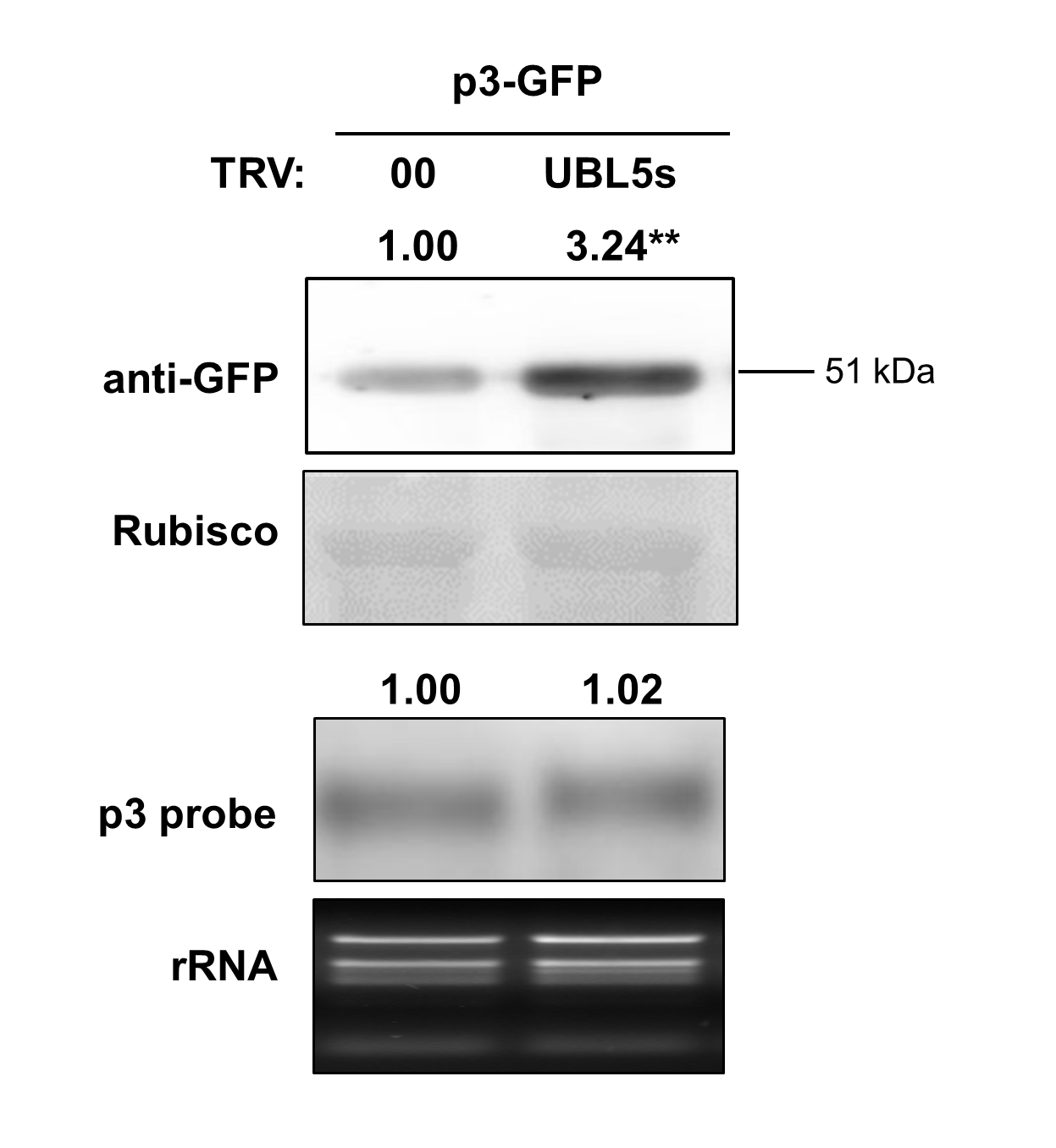

Supplement: S9 Fig — p3-GFP was expressed in NbUBL5s-silenced leaves or non-silenced leaves by infiltration for 3 days, and then the samples were collected for analysis. The results show that p3-GFP protein accumulated more in silenced than in non-silenced leaves. Results are from three biological replicates. Band intensity in blots was calculated by ImageJ based on three replicates. A two-sample unequal variance directional t test was used to test the significance of the difference (**, p value<0.01). (TIF) [file ppat.1008780.s009.TIF]

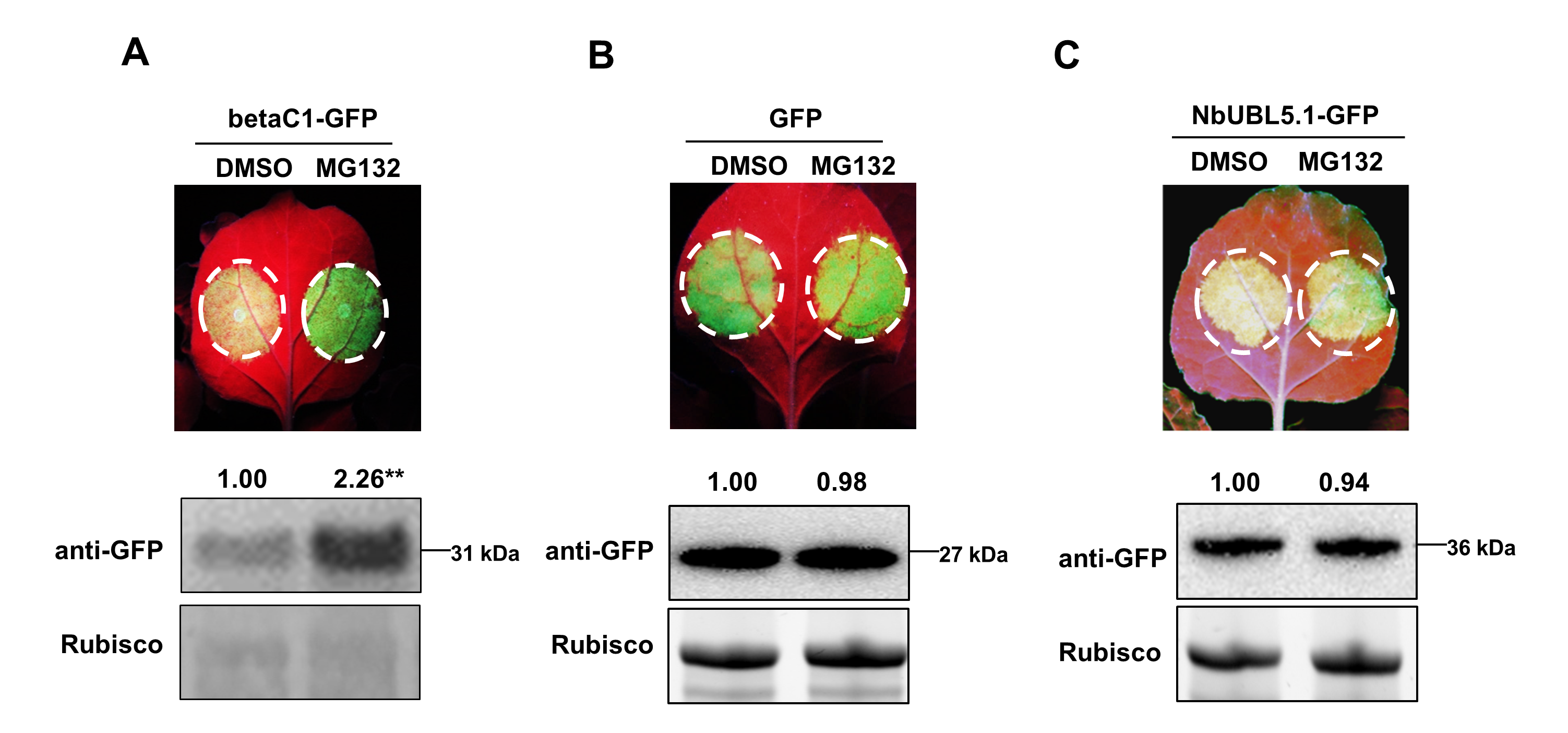

Supplement: S10 Fig — A and B. MG132 treatment inhibited the degradation of tomato yellow leaf curl China virus (TYLCCNV) βC1 protein that was reported to be degraded through the 26S proteasome system (A), but had no effect on the accumulation of GFP (B), showing that MG132 treatment had worked. C. MG132 treatment had no effect on accumulation of NbUBL5.1. Results are from three biological replicates. Band intensity in blots was calculated by ImageJ based on three replicates. A two-sample unequal variance directional t test was used to test the significance of the difference (**, p value<0.01). (TIF) [file ppat.1008780.s010.TIF]

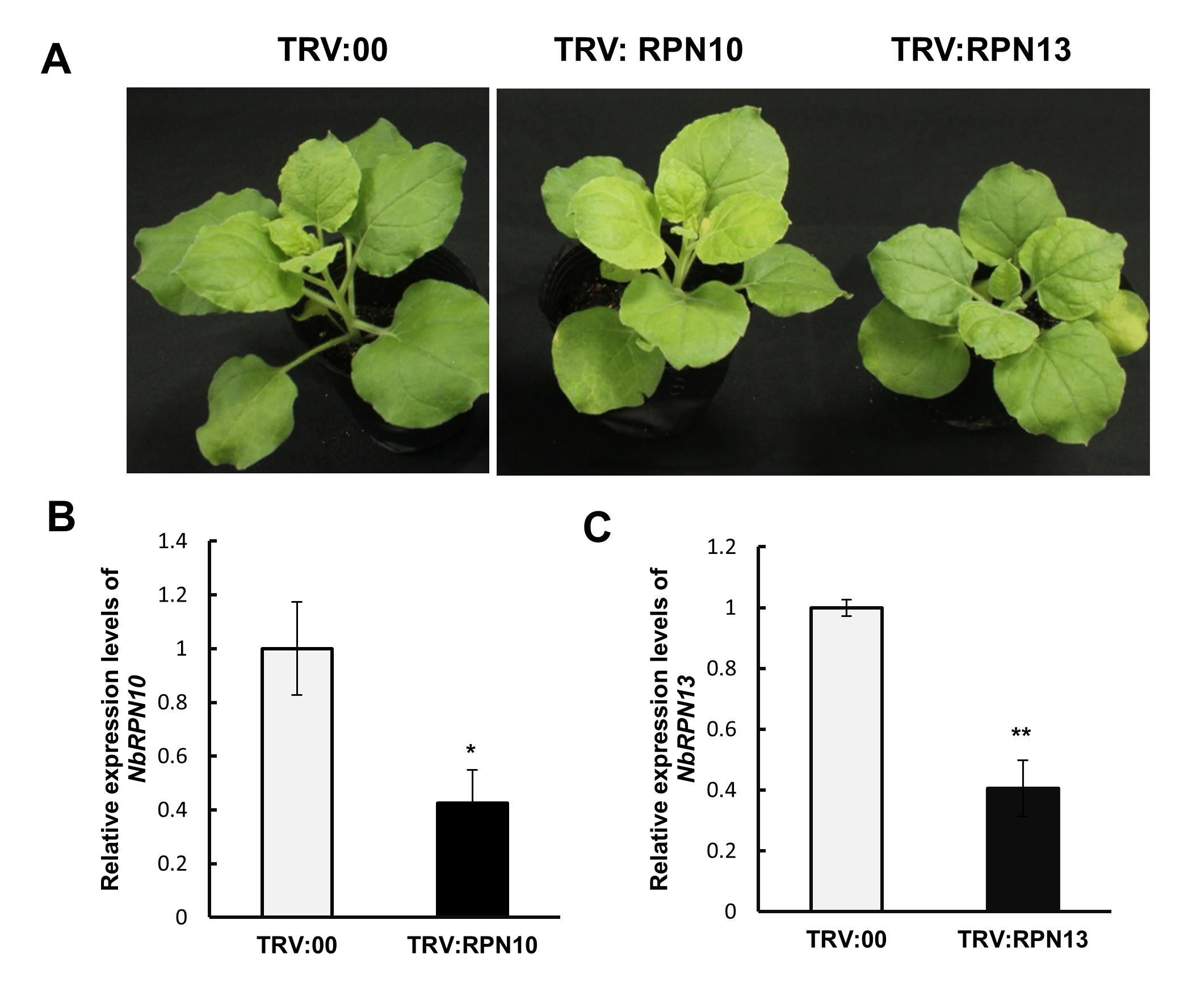

Supplement: S11 Fig — A. Plant phenotype was not greatly affected by silencing of either NbRPN10 or NbRPN13. B and C. qRT-PCR indicated the silencing of NbRPN10 and NbRPN13 in the treated plants. Results are from three biological replicates. Bars represent the standard errors of the means from three biological repeats. A two-sample unequal variance directional t test was used to test the significance of the difference (*, p value<0.05; **, p value<0.01). (TIF) [file ppat.1008780.s011.TIF]

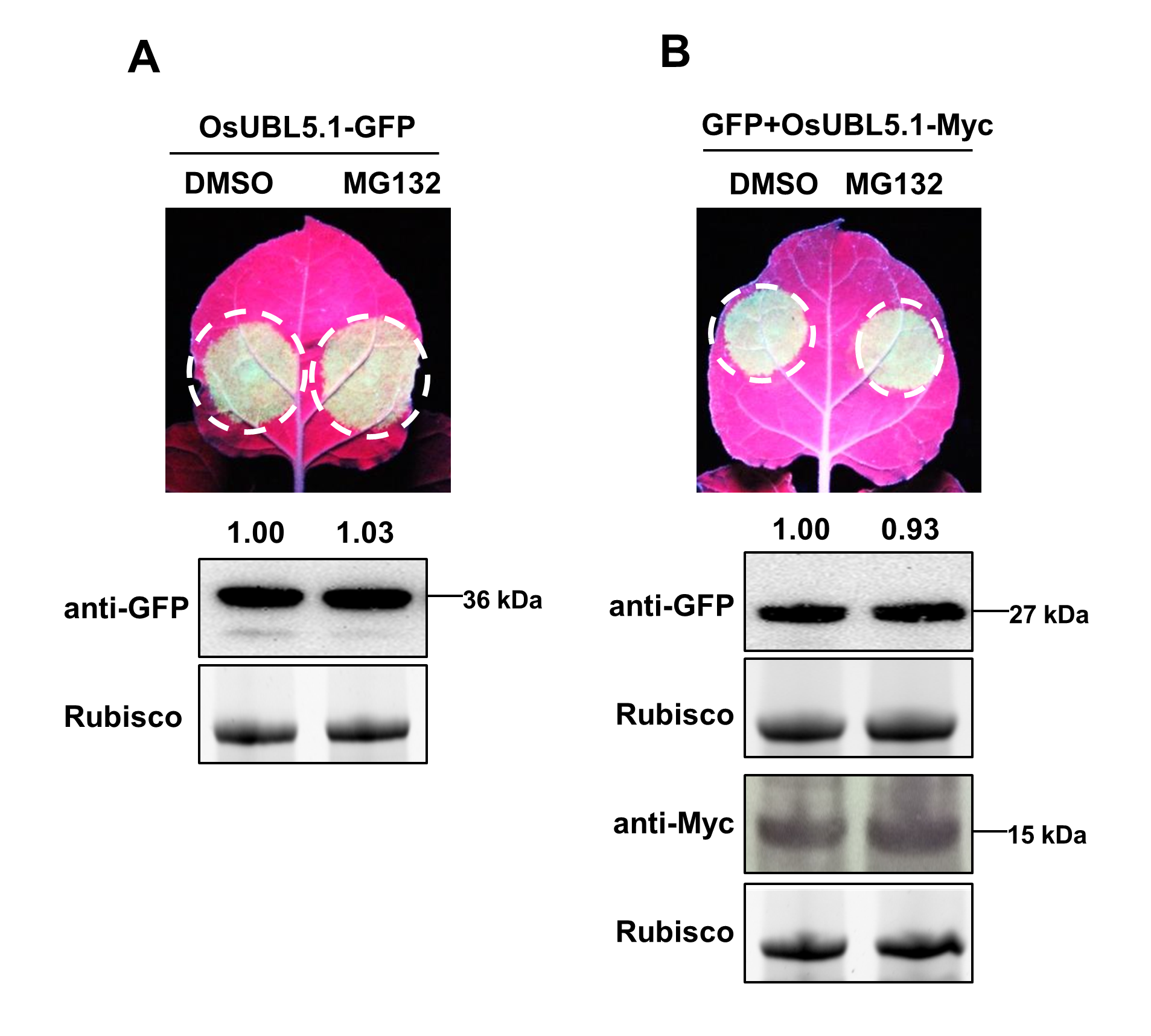

Supplement: S12 Fig — MG132 treatment had no effect on accumulation of OsUBL5.1-GFP (A) or GFP in the presence of OsUBL5.1 (B). GFP and Myc-fused proteins were detected by western blot. Results are from three biological replicates. Band intensity in blots was calculated by ImageJ based on three replicates. A two-sample unequal variance directional t test was used to test the significance of the difference. (TIF) [file ppat.1008780.s012.TIF]

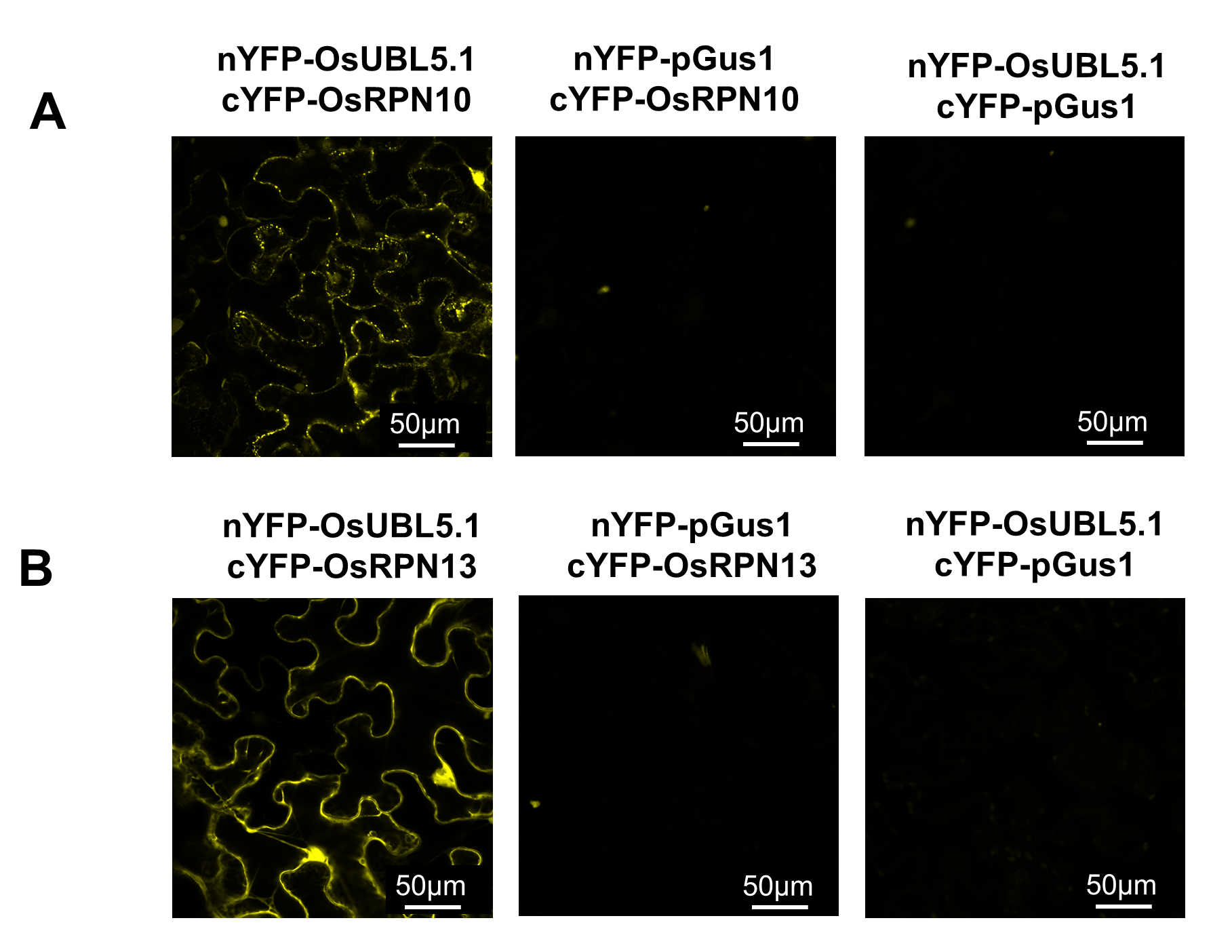

Supplement: S13 Fig — BiFC assay showing the interaction of OsUBL5.1 with OsRPN10 (A) and OsRPN13 (B). (TIF) [file ppat.1008780.s013.TIF]

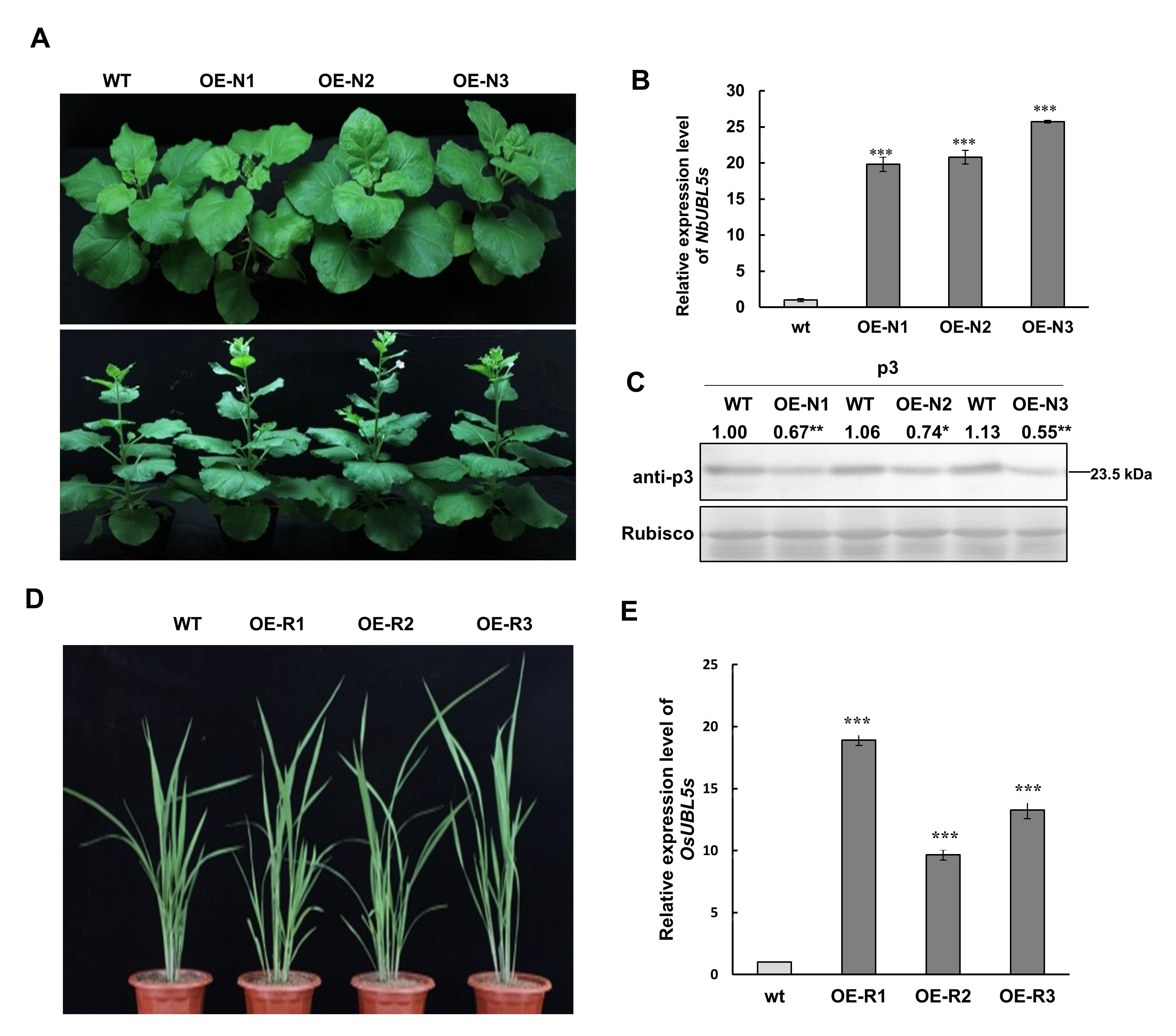

Supplement: S14 Fig — A. The phenotypes of three independent lines (OE-N1, N2 and N3) overexpressing NbUBL5.1. B. qRT-PCR showing high levels of expression of NbUBL5.1 in these lines. C. When p3 was expressed transiently in transgenic plants, its accumulation was reduced compared to that in wild type. D and E. Three independent lines (OE-R1, R2 and R3) overexpressing OsUBL5.1 were identified (D) showing high levels of expression of OsUBL5s by qRT-PCR (E). Bars represent the standard errors of the means from three biological repeats. Band intensity in blots was calculated by ImageJ based on three replicates. A two-sample unequal variance directional t test was used to test the significance of the difference (*, p value<0.05; **, p value<0.01). (TIF) [file ppat.1008780.s014.TIF]
