# Supplementary material for: Ubiquitin-Like protein 5 interacts with the silencing suppressor p3 of rice stripe virus and mediates its degradation through the 26S proteasome pathway
Source: PLoS Pathog. 2020 Aug 31;16(8):e1008780. doi: 10.1371/journal.ppat.1008780 (PMC7485977; doi:10.1371/journal.ppat.1008780)
Supplement: S1 Table — (DOCX) [file ppat.1008780.s015.docx]

**Table S1 Primers used in experiments**

| **Primer names** | **Primer sequences** |
| --- | --- |
| OsUBL5.1 LOC4330046(F) | 5'-cgacgacaagaccgtcaccAGAAGAAGAAGGGAGCGGA-3' |
| OsUBL5.1 LOC4330046(R) | 5'-gaggagaagagccgtcgATCGGCCATACTCGAAGGCA-3' |
| OsUBL5.1 LOC4330046-1(R) delete stop codon | 5'-gaggagaagagccgtcgGTCGTAGTAGAGCTCGAGGCC-3' |
| OsUBL5.2 LOC4349755(F) | 5'-cgacgacaagaccgtcaccATGATCGAGGTGGTGCT-3' |
| OsUBL5.2 LOC4349755(R) | 5'-gaggagaagagccgtcgCTAGTTGTAGTAGAGCTCGAGGCC-3' |
| OsUBL5.2 LOC4349755-1(R) delete stop codon | 5'-gaggagaagagccgtcgGTTGTAGTAGAGCTCGAGGCC-3' |
| OsUBL5(F) for qPCR | 5'-CGAGAAGATCCGCATCCAGA-3' |
| OsUBL5(R) for qPCR | 5'-CTTGAGGGTGATGTGGTCCT-3' |
| Osactin(F) for qPCR | 5'-AATGGTCAAGGCAGGTTTTG-3' |
| Osactin(R) for qPCR | 5'-ACGTGGTCTCCCTACAATGC-3' |
| Niben1028UBL5.1(F) | 5'-cgacgacaagaccgtcaccATGATAGAGGTGGTGTTGAA-3' |
| Niben1028UBL5.1(R) | 5'-gaggagaagagccgtcgTTAGTTGTAGTAGAGCTCAAG-3' |
| Niben017.1UBL5.2(F) | 5'-cgacgacaagaccgtcaccATGATAGAGGTAGTGTTGAAC-3' |
| Niben017.1UBL5.2(R) | 5'-gaggagaagagccgtcgTTAGTTGTAGTAGAGCTCAAGGC-3' |
| Niben011.1UBL5.3(F) | 5'-cgacgacaagaccgtcaccATGATAGAGGTGATTTTGAA-3' |
| Niben011.1UBL5.3(R) | 5'-gaggagaagagccgtcgTTAGTTGTAGTAAAGCTCAAGGC-3' |
| Niben1028UBL5.1(R)-1 delete stop codon | 5'-gaggagaagagccgtcgGTTGTAGTAGAGCTCAAG-3' |
| Niben017.1UBL5.2(R)-1 delete stop codon | 5'-gaggagaagagccgtcgGTTGTAGTAGAGCTCAAGGC-3' |
| Niben011.1UBL5.3(R)-1 delete stop codon | 5'-gaggagaagagccgtcgGTTGTAGTAAAGCTCAAGGC-3' |
| UBL5(F) for VIGS | 5'-cgacgacaagaccgtcaccTGACACCATCGGCGACTT-3' |
| UBL5(R) for VIGS | 5'-gaggagaagagccgtcgTTAGTTGTAGTAGAGCTCAAGGC-3' |
| UBL5(F) for qPCR | 5'-GGGGAAGAAGGTGAAGGTGA-3' |
| UBL5(R) for qPCR | 5'-TGTACCAGTCTGAGCAGCAA-3' |
| Nbactin(F) for qPCR | 5'-CACACTGGAGTGATGGTTGG-3' |
| Nbactin(R) for qPCR | 5'-GGTGTGGTGCCAAATCTTCT-3' |
| Nb1028UBL5.1F(N7-A) | 5'-cgacgacaagaccgtcaccATGATAGAGGTGGTGTTGGCAGAT-3' |
| Nb1028UBL5.1F(R9-A) | 5'-cgacgacaagaccgtcaccATGATAGAGGTGGTGTTGAACGATGCATT-3' |
| Nb1028UBL5.1F(K12-A) | 5'-cgacgacaagaccgtcaccATGATAGAGGTGGTGTTGAACGATCGATTGGGGGCAA-3' |
| Nb1028UBL5.1F(K13-A) | 5'-cgacgacaagaccgtcaccATGATAGAGGTGGTGTTGAACGATCGATTGGGGAAGGCA-3' |
| Nb1028UBL5.1F(K17-A) | 5'-cgacgacaagaccgtcaccATGATAGAGGTGGTGTTGAACGATCGATTGGGGAAGAAGGTGAAGGTGGCA-3' |
| Nb1028UBL5.1F(D22-A) | 5'-cgacgacaagaccgtcaccATGATAGAGGTGGTGTTGAACGATCGATTGGGGAAGAAGGTGAAGGTGAAGTGCAACGAGGATGCA-3' |
| Nb1028UBL5.1R(63H-A) | 5'-gaggagaagagccgtcgGTTGTAGTAGAGCTCAAGGCCCATGCCATCTGCAA-3' |
| Nb1028UBL5.1R(64D-64A) | 5'-gaggagaagagccgtcgGTTGTAGTAGAGCTCAAGGCCCATGCCTGCGTGAATTTC-3' |
| Nb1028UBL5.1R(69E-A) | 5'-gaggagaagagccgtcgGTTGTAGTAGAGTGCAAGGCCCA-3' |
| Nb1028UBL5.1R(7172YY-7172AA) | 5'-gaggagaagagccgtcgGTTTGCTGCGAGCTCAAGGCCCAT-3' |
| TYLCCNB-βC1(F) | 5'-cgacgacaagaccgtcaccATGACTATCAAATACAA-3' |
| TYLCCNB-βC1(R) | 5'-gaggagaagagccgtcgTACATCTGAATTTGTAAATAC-3' |
| pGus.1(F) | 5'-cgacgacaagaccgtcaccATGTTACGTCCTGTAGAAAC-3' |
| pGus.1(R) | 5'-gaggagaagagccgtcgTTATCGAATCCTTTGCCA-3' |
| pGus.2(R) | 5'-gaggagaagagccgtcgAATAACATACGGCGTGACA-3' |
| pGus.3(R) | 5'-gaggagaagagccgtcgCGCGTGGTTACAGTCTT-3' |
| Hygromycin(F) for qPCR | 5'-ATTTCGGCTCCAACAATGTC-3' |
| Hygromycin(R) for qPCR | 5'-GATGTTGGCGACCTCGTATT-3' |
| TRV RNA1(F) for probe | 5'-CAGTCTATACACAGAAACAGA-3' |
| TRV RNA1(R) for probe | 5'-GACGTGTGTACTCAAGGGTT-3' |
| NbRPN10(F) | 5'-cgacgacaagaccgtcaccATGGTGCTCGAGGCGACAAT-3' |
| NbRPN10(R) for VIGS and full-length | 5'-gaggagaagagccgtcgCTTCTTCTCTTCTTTCTGTTCC-3' |
| OsRPN10(F) | 5'-cgacgacaagaccgtcaccATGGTGCTCGAGGCGAC-3' |
| OsRPN10(R) | 5'-gaggagaagagccgtcgTTTCTTCTCATCTTCTGGCTTGT-3' |
| NbRPN10(F) for VIGS | 5'-cgacgacaagaccgtcaccTTTCAATGGAGGAGGAAA-3' |
| NbRPN10(F) for qPCR | 5'-TAATTTGGATCCTGAACTTG-3' |
| NbRPN10(R) for qPCR | 5'-TCTCCTTTCTCTTGTGTAGC-3' |
| NbRPN13(F) | 5'-cgacgacaagaccgtcaccATGGAATTTCGTGCCGGTAA-3' |
| NbRPN13(R) | 5'-gaggagaagagccgtcgTGATCCCATGGATGAAGGCCAC-3' |
| OsRPN13(F) | 5'-cgacgacaagaccgtcaccGTAGCCACTCTTTAATCA-3' |
| OsRPN13(R) | 5'-gaggagaagagccgtcgACTCTCATCCATGGGATCAT-3' |
| NbRPN13(F) for VIGS | 5'-cgacgacaagaccgtcaccATGGAGTTCCCTGGTGAAGA-3' |
| NbRPN13(R) for VIGS | 5'-gaggagaagagccgtcgTGGGATCAATTCCAAACTGA-3' |
| NbRPN13(F) for qPCR | 5'-CTGACAATGATTCACAAATAT-3' |
| NbRPN13(R) for qPCR | 5'-CTTCCTCTACCATATCTTCAG-3' |
| RSV p3(F) | 5'-cgacgacaagaccgtcaccATGAACGTGTTCACATCGT-3' |
| RSV p3(R) | 5'-gaggagaagagccgtcgCAGCACAGCTGGAGAGCTG-3' |
| RSV CP(F) for probe | 5'-ATGGGCACCAACAAGCCAG-3' |
| RSV CP(R) for probe | 5'-GTCATCTGCACCTTCTGCCTC-3' |
| RSV p3(F) for qPCR | 5'-TGTCAAGGGATTTCCTCCAG-3' |
| RSV p3(R) for qPCR | 5'-ACAGGCTTTCCATCATGGTC-3' |
| mGFP1(F) for probe | 5'-TCCTATCATTATCCTCGGCC-3' |
| mGFP1(R) for probe | 5'-TAAGTTTTCCGTATGTTGCATC-3' |
| mGFP2(F) for probe | 5'-CCCTTAAATTTATTTGCACTACT-3' |
| mGFP2(R) for probe | 5'-CGTATCCCTCAGGCATGGC-3' |
| mGFP3(F) for probe | 5'-TGCAGGAGAGGACCATCTTC-3' |
| mGFP3(R) for probe | 5'-ATTCCAACTTGTGGCCGAGG-3' |
| mGFP4(F) for probe | 5'-ACAACTACAACTCCCACAAC-3' |
| mGFP4(R) for probe | 5'-ACAGGGCCATCGCCAATTG-3' |
| siNbUBL5.1(BamHI/NcoI) | 5'-ggatcccatggATGATAGAGGTGGTGTTGAA-3' |
| siNbUBL5.1(XbaI/XhoI) | 5'-tctagactcgagGGTCCTTGTAGACATTGTACC-3' |
| siNbUBL5.1(F) for qPCR | 5'-GCTGGTTGCTGCTCAGACTG-3' |
| siNbUBL5.1(R) for qPCR | 5'-GTAGAGCTCAAGGCCCATGC-3' |
| siGus(BamHI/NcoI) | 5'-ggatcccatggATGTTACGTCCTGTAGAAA-3' |
| siGus(XbaI/XhoI) | 5'-tctagactcgagGATCGTTAAAACTGCCTGG-3' |
| TBSV P19(F) for full-length | 5'-cgacgacaagaccgtcaccATGGAACGAGCTATACAAG-3' |
| TBSV P19(R) for full-length | 5'-gaggagaagagccgtcgCTCGCTTTCTTTTTCGAAGG-3' |
| TuMV HC-Pro(F) for full-length | 5'-cgacgacaagaccgtcaccATGAGTGCAGCAGGAGC-3' |
| TuMV HC-Pro(R) for full-length | 5'-gaggagaagagccgtcgTCCAACGCGGTAGTGTTTCAAG-3' |
| CMV 2b(F) for full-length | 5'-cgacgacaagaccgtcaccATGGAATTGAACGAAGGCG-3' |
| CMV 2b(R) for full-length | 5'-gaggagaagagccgtcgAAACGACCCTTCCGCCCACT-3' |
